# Silencing of the *PHLDA1* leads to global proteome changes and differentiation pathways of human neuroblastoma cells

**DOI:** 10.3389/fphar.2024.1351536

**Published:** 2024-03-01

**Authors:** Beata Bugara, Małgorzata Durbas, Maja Kudrycka, Agata Malinowska, Irena Horwacik, Hanna Rokita

**Affiliations:** ^1^ Laboratory of Molecular Genetics and Virology, Faculty of Biochemistry, Biophysics and Biotechnology, Jagiellonian University, Kraków, Poland; ^2^ Doctoral School of Exact and Natural Sciences, Jagiellonian University, Kraków, Poland; ^3^ Mass Spectrometry Laboratory, Institute of Biochemistry and Biophysics, Polish Academy of Sciences, Warsaw, Poland

**Keywords:** GD2 ganglioside-binding antibody, human neuroblastoma cells, *PHLDA1* silencing, mass spectrometry, AUTS2

## Abstract

Neuroblastoma (NB) is the most common extracranial pediatric solid tumor originating from the abnormal development of cells of the sympathoadrenal lineage of the neural crest. Targeting GD2 ganglioside (GD2), a glycolipid expressed on neuroblastoma cells, with GD2 ganglioside-recognizing antibodies affects several pivotal signaling routes that drive or influence the malignant phenotype of the cells. Previously performed gene expression profiling helped us to identify the *PHLDA1* (pleckstrin homology-like domain family A member 1) gene as the most upregulated gene in the IMR-32 human neuroblastoma cells treated with the mouse 14G2a monoclonal antibody. Mass spectrometry-based proteomic analyses were applied to better characterize a role of PHLDA1 protein in the response of neuroblastoma cells to chimeric ch14.18/CHO antibody. Additionally, global protein expression profile analysis in the IMR-32 cell line with *PHLDA1* silencing revealed the increase in biological functions of mitochondria, accompanied by differentiation-like phenotype of the cells. Moreover, mass spectrometry analysis of the proteins co-immunoprecipitated using anti-PHLDA1-specific antibody, selected a group of possible PHLDA1 binding partners. Also, a more detailed analysis suggested that PHLDA1 interacts with the DCAF7/AUTS2 complex, a key component of neuronal differentiation *in vitro*. Importantly, our results indicate that *PHLDA1* silencing enhances the EGF receptor signaling pathway and combinatory treatment of gefitinib and ch14.18/CHO antibodies might be beneficial for neuroblastoma patients. Data are available via ProteomeXchange with the identifier PXD044319.

## 1 Introduction

Neuroblastoma is an embryonal malignancy of neuroectodermal origin, characterized by an impaired neuronal differentiation (Louis and Shohet, 2015). The International Neuroblastoma Risk Group Staging System regards tumor differentiation grade, histological category, stage, age, *MYCN* amplification status, DNA ploidy, and chromosome 11q status as the most important prognostic features (Cohn et al., 2009). Targeting GD2 ganglioside (a glycolipid over-expressed on neuroblastoma cells) with the therapeutic antibodies constitutes now an important way to treat chemotherapy-resistant form of high-risk neuroblastoma in children. Our *in vitro* studies showed that treating of IMR-32 neuroblastoma cells with 14G2a monoclonal antibody causes a significant decrease in cell viability and proliferation and an increase in apoptotic cell death confirmed by activation of caspase 3 (Kowalczyk et al., 2009). Moreover, a decrease of aurora kinases protein level and the phosphorylation on key amino acid residues of the kinases was observed (Horwacik et al., 2013). The effects were accompanied by a decreased survival of neuroblastoma cells, a decrease of the cytoplasmic level of MYCN oncogene, an increase in levels of P53 and downregulation of the AKT/mTOR network (Horwacik et al., 2013; Durbas et al., 2015). Gene expression profiling showed that *PHLDA1* is the most upregulated gene in the studied human neuroblastoma cells (Horwacik et al., 2015).

Some functions of *PHLDA1* in cancer are already indicated by other studies. However, its roles remain controversial due to described pro- and anti-cancer properties in various types of cancer. PHLDA1 was proposed as a marker of epithelial and follicular stem cells that contributes to the tumorigenesis of the intestine (Sakthianandeswaren et al., 2011; Sellheyer and Krahl, 2011). Similar properties of PHLDA1 were proposed by Kastrati et al. in ER + mammospheres formation of breast cancer (Kastrati et al., 2015). Coutinho-Camillo et al. reported association of PHLDA1 and PAR-4 with cases of clinically advanced oral squamous cell carcinomas (Coutinho-Camillo et al., 2013). Xu et al. reported that PHLDA1 is required for resistance to oxidative stress-induced cell death in ovarian cancer cells (Xu et al., 2021). Finally, Liu et al. showed that PHLDA1 promotes glioma growth *in vivo* (Liu et al., 2019). On the other hand, PHLDA1 downregulation was shown to induce chemoresistance of breast cancer (Fearon et al., 2018). Li et al. suggest that PHLDA1 might have key inhibitory functions in ErbB2-driven lung and breast cancer cells (Li et al., 2014). Additionally, in a model of apoptosis induced by oxidative stress in mouse embryonic fibroblasts and apoptosis induced with hydrogen sulfide in an oral cancer cell line Ca9-22, the lack of PHLDA1 was linked to activation of caspase 3. This suggests that the protein is a suppressor of apoptosis (Park et al., 2013; Murata et al., 2014). Based on the data gathered above, it can be concluded that PHLDA1 is a multifaceted protein with context-dependent functions in cancer.

Previously, to investigate the role of PHLDA1 in IMR-32 cells, we characterized effects of the *PHLDA1* gene silencing using lentiviral vectors (Durbas et al., 2016). IMR-32 cells with stable downregulation of *PHLDA1* showed enhanced cellular ATP levels and an increase in mitochondrial membrane potential as compared to control cells. Most importantly, *PHLDA1*-silenced cells were less susceptible to apoptosis and showed an increase in aurora A kinase expression as well as its activating phosphorylation at Thr288. Additionally, downregulation of *PHLDA1* caused a significant decrease in the cell cycle inhibitor, CDKN1A (P21) and an increase in expression of TRKB that are among other markers of poor prognosis in neuroblastoma. Therefore, our study has shed a new light on functions of PHLDA1 in the neuroblastoma cells, suggesting its role as a pro-apoptotic protein (Durbas et al., 2016).

In the present study, we investigated the functional relevance of PHLDA1 in IMR-32 cells in a broader context by using a proteomic-based approach. Using publicly available data sets, we have also assessed clinical relevance of PHLDA1, and proteins directly or indirectly regulated by PHLDA1. We showed that high level of expression of *PHLDA1* positively correlates with survival of *MYCN*-amplified subset of neuroblastoma patients, but proteins regulated by PHLDA1 exhibit both positive and negative correlation with survival of neuroblastoma patients. Our semi-quantitative proteome analysis indicates that ch14.18/CHO-antibody-treatment alters PHLDA1 interactome in IMR-32 cells and we confirmed that PHLDA1 interacts directly with the DCAF7/AUTS2 complex, described to be crucial for neuronal differentiation. We show that *PHLDA1* inhibition modulates balance of two AUTS2 isoforms and leads to a global phosphoproteome changes. Furthermore, *PHLDA1* knock-down leads to differentiation-like phenotype of IMR-32 cells, accompanied by a pronounced increase of proteome involved in mitochondrial processes. Our proteomic analysis revealed a strong involvement of PHLDA1 protein in the axon guidance pathway of neuronal development. Finally, we confirmed that *PHLDA1* silencing upregulates EGFR pathway and sensitizes IMR-32 cells to EGFR inhibitors. Additionally, we provided evidence that combinatory treatment of gefitinib and ch14.18/CHO antibodies might possess translational potential.

## 2 Materials and methods

### 2.1 Cell culture

IMR-32 human neuroblastoma cells (ATCC, United States, CCL-127) were cultured in Eagle’s minimum essential medium (EMEM) supplemented with 10% fetal bovine serum, 1% non-essential amino acid solution, 1 mM sodium pyruvate and 50 μg/mL gentamicin at 37 °C in a 5% CO_2_ atmosphere.

### 2.2 Co-immunoprecipitation of PHLDA1

IMR-32 cells, PBS- and ch14.18/CHO-treated (Qarziba, EUSA Pharma, Hemel Hempstead, United Kingdom), were seeded into 6-well plates (1 × 10^6^ per well) for 48 h in 5 mL of complete medium. Then cells were lysed with buffer containing 50 mM Tris-HCl pH 7.6, 150 mM NaCl, 0.5% NP-40 supplemented with protease inhibitors (cat. no. P8340, Sigma-Aldrich, Saint Louis, United States) for 15 min on ice. Samples were centrifuged at 16,000 × g for 15 min at 4°C, and supernatants were transferred to new tubes for further analysis. Protein concentration was measured using the Bicinchoninic Acid assay (B9643, Sigma-Aldrich). 2 mg of total protein extracts were used for pre-clearing to remove human heavy and light chains of ch14.18/CHO antibodies (Abs) internalized upon treatment. Pre-clearing of lysates was performed using magnetic Dynabeads™ Protein G (cat. no. 10003D, Invitrogen, Carlsbad, United States) for 1 h at 4°C on a roller. Then, pre-cleared lysate was split in two (a half for IP using anti-PHLDA1, the other half for IP with isotypic control Ab) and transferred to new tubes, while Dynabeads™ Protein G was discarded. Prepared lysates were used for immunoprecipitation performed using the Dynabeads™ Protein G Immunoprecipitation Kit (cat. no. 10007D, Invitrogen, Carlsbad, United States), according to the manufacturer’s protocol. Shortly, mouse anti-PHLDA1 Abs (sc-23866, Santa Cruz Biotechnology, Dallas, United States) and isotypic control mouse IgG2a Abs (sc-3878, Santa Cruz Biotechnology) were diluted in PBS-Tween-20 (0.1%), added to magnetic beads and incubated for 30 min on the rotating platform. Following washing of beads, pre-cleared cell lysates containing antigen of interest (Ag) were added to beads and resuspended. Samples were incubated overnight at 4°C on a roller. The following day, supernatants were removed, sampled, and beads-Abs-Ag complexes were washed 2 times in buffer containing 50 mM Tris-HCl pH 7.6, 150 mM NaCl and 0.5% NP-40 and last 3 times in buffer containing 50 mM Tris-HCl pH 7.6 and 150 mM NaCl. Then, beads were collected and used for mass spectrometry analysis. A small portion of beads-Abs-Ag complexes was sampled before the last washing step to proceed with the elution step. For DCAF7 and AUTS2 detection in Co-IP experiments, all the sample was subjected to elution. Elution buffer was added (100 mM Tris-HCl pH 6.8, 4% SDS, 10 mM EDTA, 100 mM DTT) to beads-Abs-Ag complexes and samples were heated for 5 min at 65°C. After removing beads, samples were loaded on a polyacrylamide gel and the SDS PAGE electrophoresis was performed.

### 2.3 Mass spectrometry analysis of proteins co-immunoprecipitated with PHLDA1

To each sample, 20 µL of 100 mM NH_4_HCO_3_ and 2.5 µL of 200 mM TCEP [tris(2-carboxyethyl) phosphine] were added; samples were vortexed and placed in a horizontal shaker (10,000 rpm) at room temperature for 30 min. Subsequently, 2 µL of MMTS (methyl methanethiosulphonate) were added and samples were shaken for 20 min at room temperature. Trypsin (Promega) was dissolved in 100 mM NH_4_HCO_3_, to a final enzyme concentration of 0.02 g/L and 50 µL of the solution was added to each sample. Samples were incubated with shaking at 37°C overnight. Next, samples were acidified with 10 µL of 5% trifluoroacetic acid (TFA). MS analysis was performed by LC-MS in the Laboratory of Mass Spectrometry (IBB PAS, Warsaw) using a nanoAcquity UPLC system (Waters, Milford, Massachusetts, United States) coupled to an Orbitrap QExactive or Orbitrap Elite mass spectrometer (Thermo Fisher Scientific, Waltham, Massachusetts, United States). The mass spectrometer was operated in the data dependent MS2 mode, and data were acquired in the m/z range of 300–2000. MS1 resolution was 70,000 or 30,000, with AGC target 1e6. For MS2, resolution was set to 35,000 or 15,000, AGC target 5e5 or 2e5, with fragmentation of 12 or 10 ions between scans. Peptides were separated by a 160 min linear gradient with 0.1% formic acid in water as phase A and 0.1% formic acid in acetonitrile as phase B on RP-C18 column (BEH130 C18 column, 75 µm ID., 25 cm long, Waters, Milford, Massachusetts, United States). The measurement of each sample was preceded by washing runs to avoid cross-contamination. Data were analyzed with the Max-Quant (Version 2.0.1.0) platform. The human reference proteome database from UniProt was used (version 2022_01, 79,502 entries) with contaminants included. Initial precision for parent and fragment ion mass was set to 20 ppm, with subsequent re-calibration performed by MaxQuant. Variable modifications were set for methionine oxidation, acetyl N-term, constant modification: methylation on cysteines. Other parameters were as follows: PSM and protein FDR – 0.01 (with reversed database), enzyme–Trypsin/P, missed cleavages - 2.

The list of identified proteins with MaxQuant software was further analyzed in Perseus software (version 1.6.15) in a semi-quantitative analysis to determine which proteins co-purify with the bait protein PHLDA1. Four experimental conditions in two repetitions were studied, including two negative controls. After data cleanup (proteins identified by site, from reversed database, contaminants), missing values were inputed by constant “1”. Relative protein intensity sample/control (Supplementary Table S1. columns Q-T) was used as a quantitative value to identify proteins enriched in one or both repetitions (Supplementary Table S1. columns J-L).

### 2.4 *PHLDA1* silencing

IMR-32 cells (1 × 10^6^ cells per well of a 6-well plate) were seeded 24 h before transfection. Then medium was replaced with 1.8 mL of complete medium followed by adding 0.2 mL of transfecting mixture containing 3 µg of DNA (plasmid expressing shRNA toward PHLDA1, cat. no. HSH005478-nH1-a, GeneCopoeia, Rockville, United States), 6 µL of Jet-PRIME reagent (cat. no. 114-07, Polyplus, Illkirch, France) and Jet-PRIME buffer, prepared according to the manufacturer’s instructions. Additionally, cells were transfected with a plasmid carrying non-targeting control shRNA (cat. no. CSHCTR001-nH1, GeneCopoeia, Rockville, United States) and a plasmid expressing control shRNA and eGFP (cat. no. CSHCTR001-CH1, GeneCopoeia, Rockville, United States) as a control of transfection efficiency. One well was used as the non-transduced control. The plate was centrifuged for 15 min, 1,000 x g at room temperature. After 4 h of incubation, medium was replaced. After 5 days of cell culture propagation, puromycin (cat. no. sc-108071, Santa-Cruz Biotechnology, Dallas, TX, United States) was added at the concentration of 0.25 μg/mL to select cells stably expressing shRNA. Puromycin concentration was lowered to 0.05 μg/mL when a small number of cells remained attached to the bottom of the culture flasks. Then puromycin concentration was gradually increased to 0.5 μg/mL. The ability of shRNA to inhibit *PHLDA1* gene expression was assessed by Western blot (WB) analysis.

### 2.5 The phosphoproteome profiling antibody array

*PHLDA1*-stably silenced clones of IMR-32 cells (S2 and S4), control (Mock) cells and non-transduced (WT) IMR-32 cells were prepared using the shRNA lentiviral particles as previously described (Durbas et al., 2016) and seeded into a 6-well plate (1 × 10^6^ per well). S2, S4 and Mock cells were grown in 5 mL of selection medium with addition of puromycin dihydrochloride (concentration - 0.5 μg/mL), while WT cells were cultured in 5 mL of complete medium. Following 48 h of culture, cells from the respective groups were lysed in Lysis Buffer provided from the human phospho-RTK Array Kit (cat. no. ARY001B, R&D systems, Minneapolis, United States) 300 μg of total protein extract was used for the phosphoproteome profiling antibody arrays. Detection of tyrosine kinase receptors was performed according to the manufacturer’s instruction.

### 2.6 Receptor tyrosine kinases inhibitors and antibody treatment

Wild type or genetically modified IMR-32 cells (shPHLDA1 - *PHLDA1* silenced and shCtrl - mock) were incubated on ice for 1 h and treated with tyrosine kinases inhibitors in concentrations 0.1–10 μM for gefitinib (cat. no. SML1657, Sigma-Aldrich, Saint Louis, United States) and 0.5–15 μM for lapatinib (cat. no. CDS022971, Sigma-Aldrich, Saint Louis, United States) diluted in DMSO (cat. no. D2650, Sigma-Aldrich, Saint Louis, United States) and seeded for 72 h. Final concentration of DMSO in cell cultures was 0.1%. Inhibitors were used alone or in combination with ch14.18/CHO antibodies (5 μg/mL) on ice for 1 h, then cells were incubated for 72 h in 37°C, 5% CO_2_. IC_50_ values were determined by fitting a dose response curve to the data points using non-linear regression analysis with the Excel software (Microsoft, Redmond, United States). To isolate proteins, cells were seeded on 6-well plates (1 × 10^6^/5 mL/well). Control cells were treated with DMSO (for inhibitors) and PBS (for ch14.18/CHO).

### 2.7 hEGF stimulation

IMR-32 cells (5.6 × 10^5^ cells per well of 6-well plate) were seeded for 48 h in 5 mL of complete medium. Then medium was replaced with 0.5% FBS medium for additional 24 h, followed by stimulation with recombinant hEGF (cat. no. 236-EG, R&D, Minneapolis, United States of America) at concentration of 20 ng/mL for 15 min in case of shPHLDA1 and shCtrl IMR-32 cells and 20 or 100 ng/mL for 15 min, 1 h and 24 h in unmodified cells. Control cells were treated with equivalent volume of 0.1% BSA in PBS (solvent for hEGF).

### 2.8 ATP level measurements

IMR-32 (2 × 10^4^) cells were cultured for 72 h in 100 μL of complete medium in a 96-well plate, in triplicates. Comparison of metabolic activity/viability was made by measuring cellular ATP levels using the ATPlite - luminescence ATP detection assay kit (cat. no. 6016947, Perkin-Elmer, Warszawa, Poland) according to the manufacturer’s protocol. Relative ATP levels were calculated as follows: relative ATP level = ATP level in cells treated with inhibitor or antibody/ATP level in control cells. Samples were analyzed using the Infinite M200 reader (TECAN, Männedorf, Switzerland).

### 2.9 Trypan blue viability tests

Modified IMR-32 cells: shPHLDA1 and shCtrl, were treated with EGFR inhibitors, according to the previously described procedure, and cultured at density of 2 × 10^5^ cells in 1 mL of complete medium in a 24-well plate for 72 h. Then, media above the cells were collected, adherent cells were treated with 0.125 mL of TrypLE™ (cat no. 12604021, ThermoFisher, Waltham, United States) per well and incubated for 1–2 min in 37 C. Afterwards, the TrypLE™ reagent was neutralized by addition of the previously collected media. In order to dissociate clumps, cell suspensions were submitted to a slow pipetting (35x per sample) by pushing the 1 mL tip against the bottom of the tube. Single cell suspensions were stained with trypan blue (cat. no. T8154, Sigma-Aldrich, Saint Louis, United States) in 1:1 ratio and cells were counted manually in a Bürker counting chamber.

### 2.10 Protein isolation and immunoblotting

Cells were lysed in RIPA buffer supplemented with protease (cat. no. P8340, Sigma-Aldrich, Saint Louis, United States) and phosphatase inhibitors (PhosSTOP, cat. no. 4906845001, Roche, Basel, Switzerland) or lysed with TRI-Reagent^®^ (cat. no. TR118, Lab Empire, Rzeszow, Poland). Protein concentration was measured using the Bicinchoninic Acid assay (BCA) method. Aliquots (10–60 μg of total protein) of cell lysates were used for electrophoresis in 10 or 12% polyacrylamide gels by standard SDS-PAGE procedures and electro-transferred to polyvinylidene difluoride (PVDF) membranes (Millipore Corporate, MA, United States). After the transfer, blots were blocked with 5% nonfat dry milk in TBS, 0.1% Tween 20 for 1 h. Then, blots were incubated overnight at 4°C with respective primary antibodies (Supplementary Table S8). Next, membranes were incubated with proper secondary antibodies conjugated to HRP for 1 h at RT. Protein bands were detected by chemiluminescence with a luminol reagent (cat. no. WBKLS0500, Millipore Corporate, MA, United States or cat. no RPN2105 Amersham, Cytiva, MA, United States) on ChemiDoc (Bio-Rad laboratories, Hercules, CA, United States). ImageLab software (Bio-Rad laboratories, Hercules, CA, United States) was used to visualize the protein bands. Some membranes were stripped with 0.2–0.4 M NaOH and incubated with antibodies against other proteins. GAPDH, α-tubulin and β-actin were used as reference proteins.

### 2.11 Microscopic imaging

ShPHLDA1 and shCtrl IMR-32 cells (2 × 10^4^ and 4 × 10^4^ cells per well of a 24-well plate) were seeded in 0.7 mL of complete medium. Microscopic observations were made after 24, 48, 72, 96 h and 7, 8, 9 days under the ×20 magnification, using the light microscope DMi1 with FLEXACAM C1 Camera (Leica Microsystems, Wetzlar, Germany). Media were replaced after 7 days from seeding.

### 2.12 Mass spectrometry analysis of proteome and post-translational modifications

ShPHLDA1 and shCtrl IMR-32 cells (1 × 10^6^ cells per well of 6-well plate) were seeded for 48 h in 5 mL of complete medium. Then cells were lysed with buffer containing 25 mM Tris-HCl pH 7.6, 150 mM NaCl, 1% sodium deoxycholate, 0.1% SDS, supplemented with protease (cat. no. P8340, Sigma-Aldrich, Saint Louis, United States) and phosphatase inhibitors (PhosSTOP, cat. no. 4906845001, Roche, Basel, Switzerland). Protein concentration was measured using Bicinchoninic Acid assay (BCA) method. 40 μg of total protein extract was precipitated by adding four times the volume of cold (−20 °C) acetone, followed by 1 h incubation in −20°C and centrifugation for 10 min at 14,000 x g at 4°C. Five biological replicates per group were submitted for MS analysis at the Mass Spectrometry Laboratory of the Institute of Biochemistry and Biophysics (PAS, Warsaw, Poland). Protein pellets were resuspended in 50 µL of 100 mM ammonium bicarbonate buffer (ABC) by 30 min vortexing and sonication. Cysteines were reduced by 1-h incubation with 50 mM tris(2-carboxyethyl) phosphine (TCEP) at 60°C followed by 30 min incubation at a room temperature with 20 mM 2-chloroacetamide (CAA). Digestion was performed at 37°C overnight with 2 µg of trypsin (Promega). After digestion, peptides were diluted to 120 µL with water and acidified to a final concentration of 0.1% formic acid (FA).

Samples were analysed using LC-MS system composed of Evosep One (Evosep Biosystems, Odense, Denmark) coupled to an Orbitrap Exploris 480 mass spectrometer (Thermo Fisher Scientific, Bremen, Germany). Samples were loaded onto disposable Evotips C18 trap columns (Evosep Biosystems, Odense, Denmark) according to the manufacturer protocol. Chromatography was carried out at a flow rate 220 nL/min using the 88 min (15 samples per day) preformed gradient on EV1106 analytical column (Dr Maisch C18 AQ 1.9 µm beads, 150 µm ID, 15 cm long, Evosep Biosystems, Odense, Denmark). Data was acquired in positive mode with a data-dependent method using the following parameters. MS1 resolution was set at 60,000 with a normalized AGC target 300%, Auto maximum inject time and a scan range of 300–1,600 m/z. For MS2 mode, resolution was set at 15,000 with a Standard normalized AGC target and top 40 precursors considered for MS/MS analysis.

Raw files were analyzed with MaxQuant platform as described in *2.3*, with modifications: activated “Match between runs” option and additional variable modifications–Phospho (STY) and GlyGly(K). Further analysis was performed in Perseus (version 1.6.15). Data were cleaned, log transformed (log2) and *t*-test with permutative FDR was performed on proteins with more than 3 quantitative values in each group to identify significantly changed proteins. Proteins absent in one of the groups were defined as proteins lacking quantitative values in one group (or having just 1 out of 5), with more than 3 quantitative values in the second group (Supplementary Table S2). Similar analysis was performed for modification sites identified in the same data (five replicates from each group), based on measured peptide intensities (Supplementary Table S3 for ubiquitination, Supplementary Table S4 for phosphorylation). Detailed protein identification and quantification data, detailed peptide data and detailed peptide fragmentation data for shPHLDA1 and shCtrl, are shown in Supplementary Table S5, Supplementary Table S6 and Supplementary Table S7, respectively. Annotated spectra are made available through Protein Prospector MS-Viewer interface https://msviewer.ucsf.edu/prospector/cgi-bin/mssearch.cgi?report_title=MS-Viewer&search_key=fhlhfe0mek&search_name=msviewer.

### 2.13 Bioinformatic analysis

Bioinformatic analysis was performed by using tools implemented in R2 (http://r2.amc.nl, http://r2platform.com) on the datasets referred to in the text as Cancer Cell Line Encyclopedia (CCLE-Broad) (Ghandi et al., 2019; Nusinow et al., 2020) Cangelosi-786 (Cangelosi et al., 2020), Kocak-649 (GSE45547) (Kocak et al., 2013), SEQC (GSE62564) (Su et al., 2014), Mixed Pediatric Pan Cancer dataset (Gröbner et al., 2018), George-12 (GSE165748) (Sengupta et al., 2022) Versteeg/Etchevers-34 (R2 internal id: ps_avgpres_gsenatgengeo34_u133p2), Mabe (GSE180514) (Mabe et al., 2022) and Mabe (GSE180515) (Mabe et al., 2022). Protein-protein interaction network analysis was prepared and visualized using STRING v10 platform (Szklarczyk et al., 2015). Gene Ontology molecular function, biological process and cellular component terms enrichment analysis were prepared using the DAVID 6.8 (Huang et al., 2009; Sherman et al., 2022) and Reactome platform (Gillespie et al., 2022). Prediction of site-specific kinase-substrate relations from phosphoproteome data was performed using the iGPS 1.0 software (Song et al., 2012). Venn diagrams were prepared using InteractiVenn platform (Heberle et al., 2015).

### 2.14 Statistical rationale

Four experimental conditions in two repetitions were studied for co-IP semi-quantitative analysis, based on sample/control ratios without further statistical steps. For shPHLDA1/shCtrl quantification, five replicates per group were employed, with two-sample *t*-test and permutation-based FDR to establish statistical significance. Data is presented as means ± SEM (a standard error of the mean). All experiments were performed in at least three independent experiments, unless stated otherwise in the figure legends. Statistical analyses for inhibitors and combined treatment experiments were performed using *t*-test and one-way or two-way analysis of variance (ANOVA) followed by Tukey’s *post hoc* comparison test to determine which values differed significantly from controls. The analyses were performed with R (R version 3.2.1 Patched) and Excel software (Microsoft, Redmond, United States). Data were considered statistically significant at *p* < 0.05 [*p*-values: *p* < 0.05 (*), *p* < 0.01 (**), *p* < 0.001 (***)].

## 3 Results

### 3.1 *PHLDA1* expression positively correlates with the survival of MYCN-amplified neuroblastoma patients

To compare the PHLDA1 protein level, between different cancer cell lines we used R2: Genomics Analysis and Visualization Platform (http://r2.amc.nl, http://r2platform.com). Analysis of Cancer Cell Line Encyclopedia proteomics dataset of 378 samples from 24 different cancer types (Nusinow et al., 2020) revealed the lowest level of PHLDA1 protein in leukemia-derived cell lines and the highest in neuroblastoma (Supplementary Figure S1A). To evaluate the correlation of *PHLDA1* expression level with survival of neuroblastoma patients we re-analyzed mRNA microarray results of Tumor Neuroblastoma SEQC dataset from 378 samples (Su et al., 2014), available on R2 platform. The analysis revealed that *PHLDA1* mRNA level positively correlates with event-free and overall survival of 92 neuroblastoma patients with *MYCN*-amplification (Supplementary Figures S1B, C).

### 3.2 Identification of PHLDA1 protein binding partners in neuroblastoma cells

**FIGURE 1:**
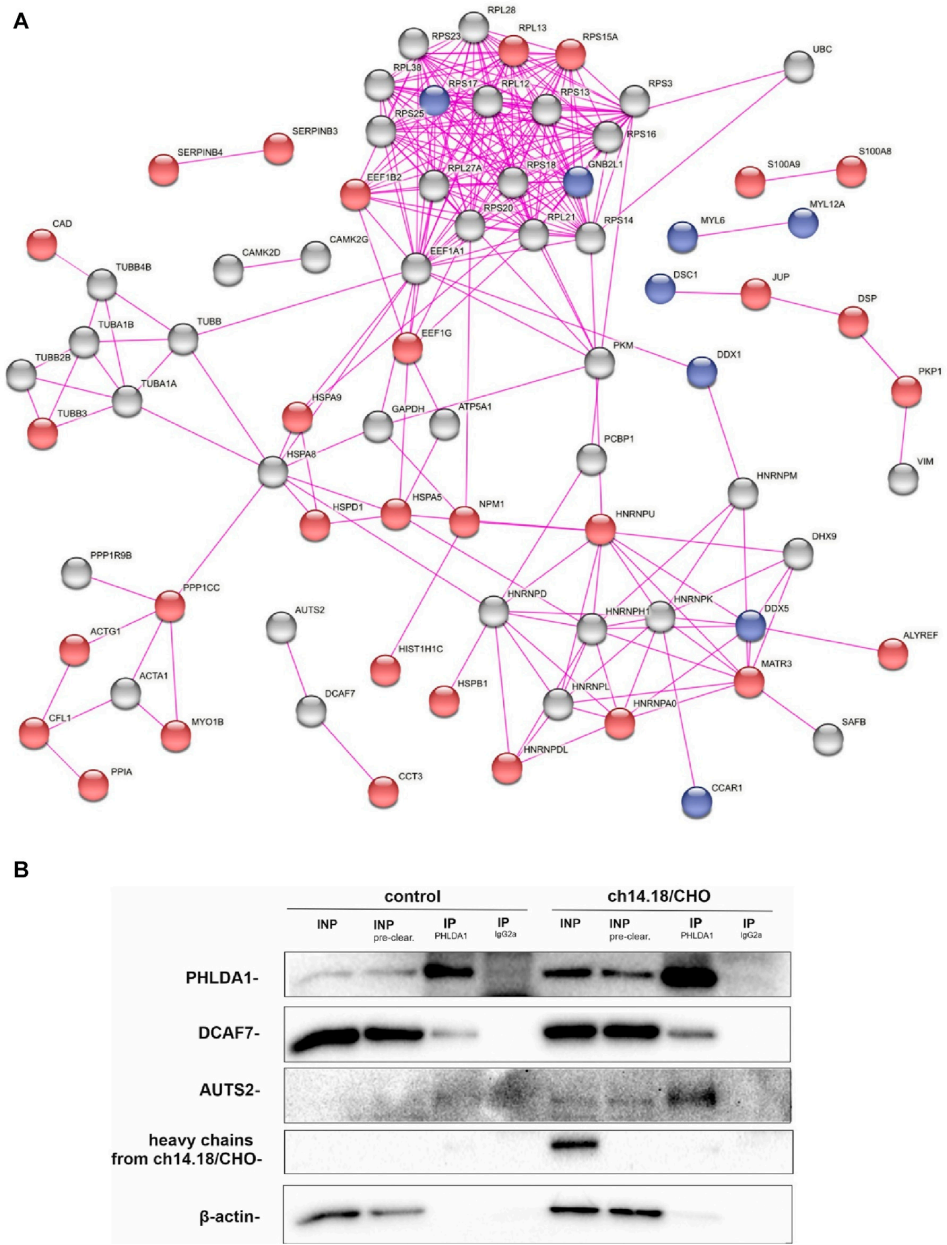
PHLDA1 directly interacts with DCAF7 and AUTS2 proteins. Control and ch14.18/CHO antibodies treated IMR-32 cells were subjected to co-IP using anti-PHLDA1 antibody. An IgG2a antibody served as the isotypic control. A subset of mass spectrometry analysis results is presented as the interaction network of PHLDA1-binding candidates previously described to exist in physical complexes, as found by STRING-DB. Proteins enriched only in control or antibodies treated cells were colored blue and red, respectively. PHLDA1 interactors enriched in both groups were colored grey. Edges indicate previously described physical interactions between proteins **(A)**. See also Supplementary Table S1. Binding proteins detected at the higher level in the isotypic control than in the test samples were regarded as contaminants. Representative Western blot analysis of three independent co-IP experiments. INP-input (10 μg), IP-immunoprecipitation (1 mg of full protein used for IP), INP - input before pre-clearing, INP pre-clear - input after pre-clearing **(B)**.

To investigate how the ch14.18/CHO-treatment affects PHLDA1 protein interactors in neuroblastoma cells, we compared potential PHLDA1 binding proteins in control- and the antibody-treated IMR-32 cells by co-immunoprecipitation, using anti-PHLDA1 antibodies followed by mass spectrometry. Efficiency of immunoprecipitation was confirmed by Western blot before mass spectrometry (Supplementary Figure S2). Overall, the analysis yielded 111 potential PHLDA1-binding partners in control- or ch14.18/CHO-treated cells (Supplementary Table S1). To extract the proteins that were previously described to exist in physical complexes we used the network analysis platform, STRING (Szklarczyk et al., 2015). The visualization indicated that the great majority (103 out of 111) of detected PHLDA1 binding candidates exist in the experimentally confirmed complexes (Figure 1A). 56 proteins were enriched in both PBS-treated and ch14.18/CHO groups, whereas after treatment with the anti-GD2 antibodies, 43 new proteins appeared, not detected in the control cells. 13 proteins that were detected in the control cells, were no longer present after the ch14.18/CHO treatment (Figure 1A, Supplementary Table S1). The signaling pathways enrichment analysis performed using Reactome platform (Gillespie et al., 2022) revealed that detected PHLDA1 binding candidates participate in plethora of pathways with significant enrichment of the antimicrobial response and signaling by Rho GTPases in ch14.18/CHO-specific PHLDA1 interactome (Supplementary Figure S3A, Supplementary Table S1). On the other hand, PHLDA1 binding candidates specific only for control cells relate most significantly to the glutamate and glutamine metabolism. Furthermore, PHLDA1 potential interactors enriched in both control and antibodies treated cells relate to the protein and RNA metabolism (Supplementary Figure S3C). Among detected binding candidates, interaction of endogenous PHLDA1 with DCAF7 and AUTS2 was confirmed by co-immunoprecipitation and immunoblotting, nevertheless we observed very low expression of endogenous AUTS2 protein in IMR-32 cells (Figure 1B).

### 3.3 Ch14.18/CHO treatment affects levels of PHLDA1 binding proteins

**FIGURE 2:**
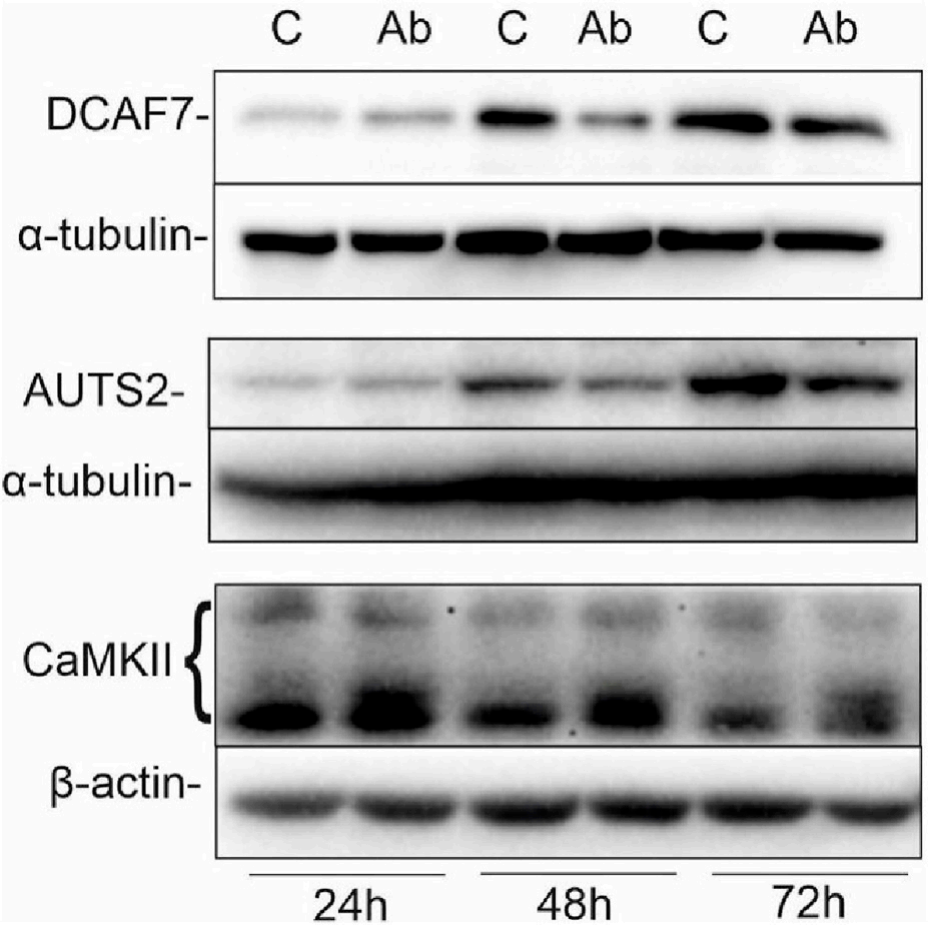
Ch14.18/CHO treatment affects levels of PHLDA1 partner proteins. IMR-32 cells were treated with 40 μg/mL ch14.18/CHO antibodies for 24, 48 and 72 h α-tubulin or β-actin were used as reference proteins. Representative immunoblots are presented. Experiments were performed at least three times. 20–60 μg protein lysate per well was used.

Previously, we have shown that targeting GD2 ganglioside in IMR-32 with 14G2a antibody strongly enhances *PHLDA1* expression (Horwacik et al., 2015). To broaden the knowledge about PHLDA1 pathways, we incubated IMR-32 cells for 24, 48 and 72 h with 40 μg/mL of ch14.18/CHO in order to show what effect this would have on selected PHLDA1 partner proteins (e.g., DCAF7 and AUTS2). Control cells were treated with equivalent volumes of PBS. Next, we collected the protein lysates and Western blot analyses were performed (Figure 2). The level of DCAF7, which was confirmed as PHLDA1 partner with two different methods in this work, decreases after 48 h of the ch14.18/CHO antibody treatment in comparison to control. Additionally, we detected a slight decrease in AUTS2 protein level after 48 and 72 h of the ch14.18/CHO antibody treatment.

### 3.4 *PHLDA1* silencing leads to differentiation-like phenotype

**FIGURE 3:**
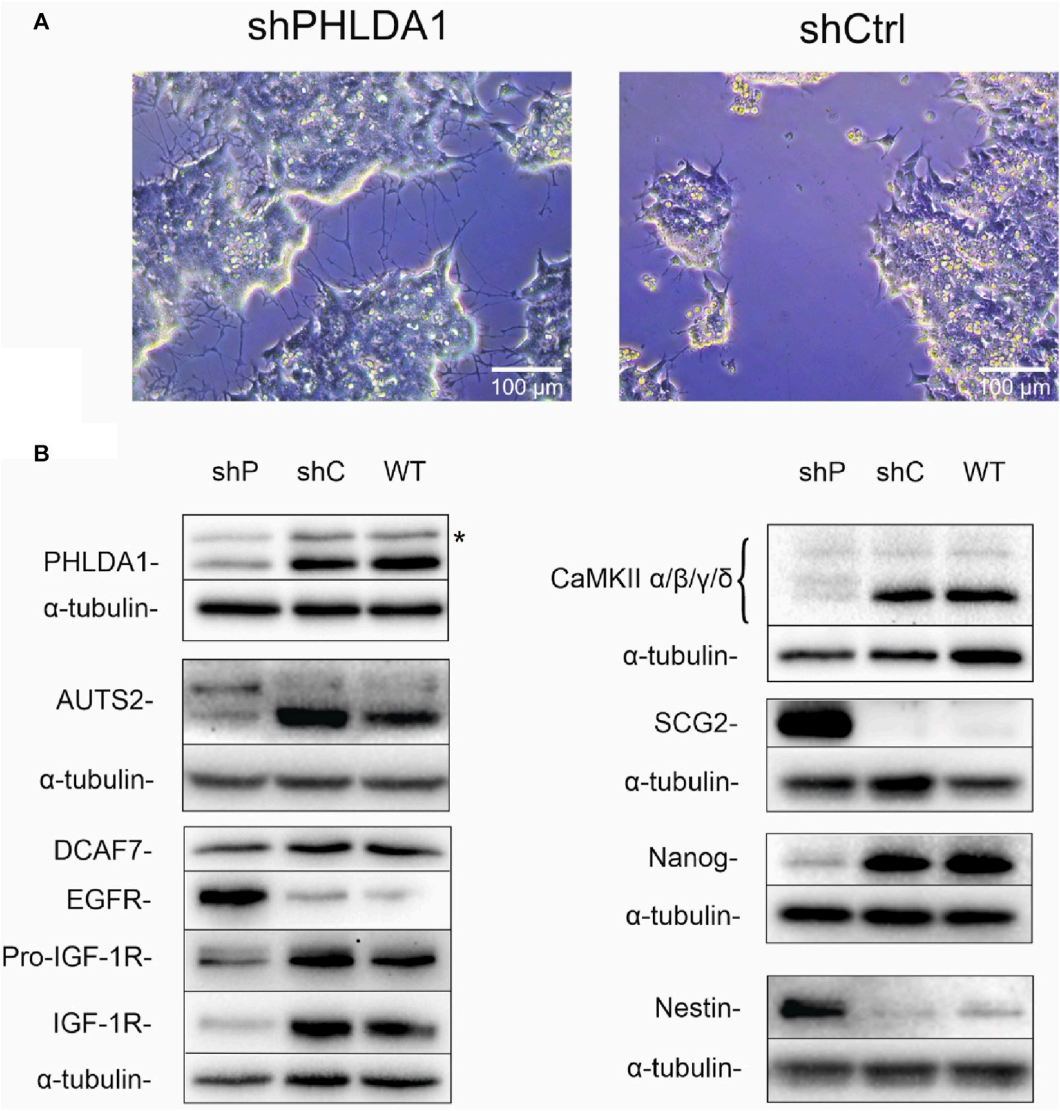
*PHLDA1*-silenced IMR-32 cells develop a differentiation-like phenotype. Stable *PHLDA1* silencing was obtained using shRNA-carrying plasmid. Microscopic observations were made on following days from seeding (See also Supplementary Figure S4). Representative images were captured 7 days from seeding of 4 × 10^4^ cells **(A)**. Cells were seeded for 48 h, lysed and Western blot analysis was performed using indicated antibodies. α-tubulin was used as a reference protein. Results are shown as representative blots of three independent experiments **(B)**. shPHLDA1 (shP) – IMR-32 cells transfected with shRNA against *PHLDA1*, shCtrl (shC)– IMR-32 cells transfected with control plasmid, WT–IMR-32 cells non-transfected with plasmid. *band of unknown origin - see also Supplementary Figure S16.

To better characterize the role of PHLDA1 in IMR-32 cells, we diminished its expression using shRNA. Microscopic observations of *PHLDA1*-silenced cells revealed that they developed differentiation-like phenotype, demonstrated by a significant neurite outgrowth in comparison to control cells (Figure 3A, Supplementary Figure S4). Furthermore, we evaluated levels of several proteins using Western blot analysis (Figure 3B, Supplementary Figure S5). *PHLDA1* silencing clearly diminishes the level of pluripotent marker Nanog and at the same time strongly upregulates Nestin, a marker of neuronal progenitor cells (Geng et al., 2022) and SCG2, a marker of neuronal differentiation into sympathetic neurons (Weiler et al., 1990). Furthermore, *PHLDA1* silencing significantly upregulates EGFR, downregulates IGF-1R and affects levels of a few of previously detected potential PHLDA1 binding partners, such as CaMKII and the aforementioned AUTS2, which together with DCAF7 was described as a member of PRC1 multiprotein complex, essential for neuronal differentiation of mouse stem cells (Wang et al., 2018). The best described molecular function of the PRC1 complex is mono-ubiquitination of histone H2A at lysine-119, necessary for repression of gene expression. Therefore, we examined the level of this modification in *PHLDA1*-silenced cells, but we did not observe significant changes in comparison to control (Supplementary Figure S5). Nevertheless, our bioinformatic analysis of datasets available on R2 platform stress out the importance of different isoforms of AUTS2 in patients with neuroblastoma (Supplementary Figures S6, S7).

### 3.5 *PHLDA1* inhibition globally upregulates mitochondrial proteome

**FIGURE 4:**
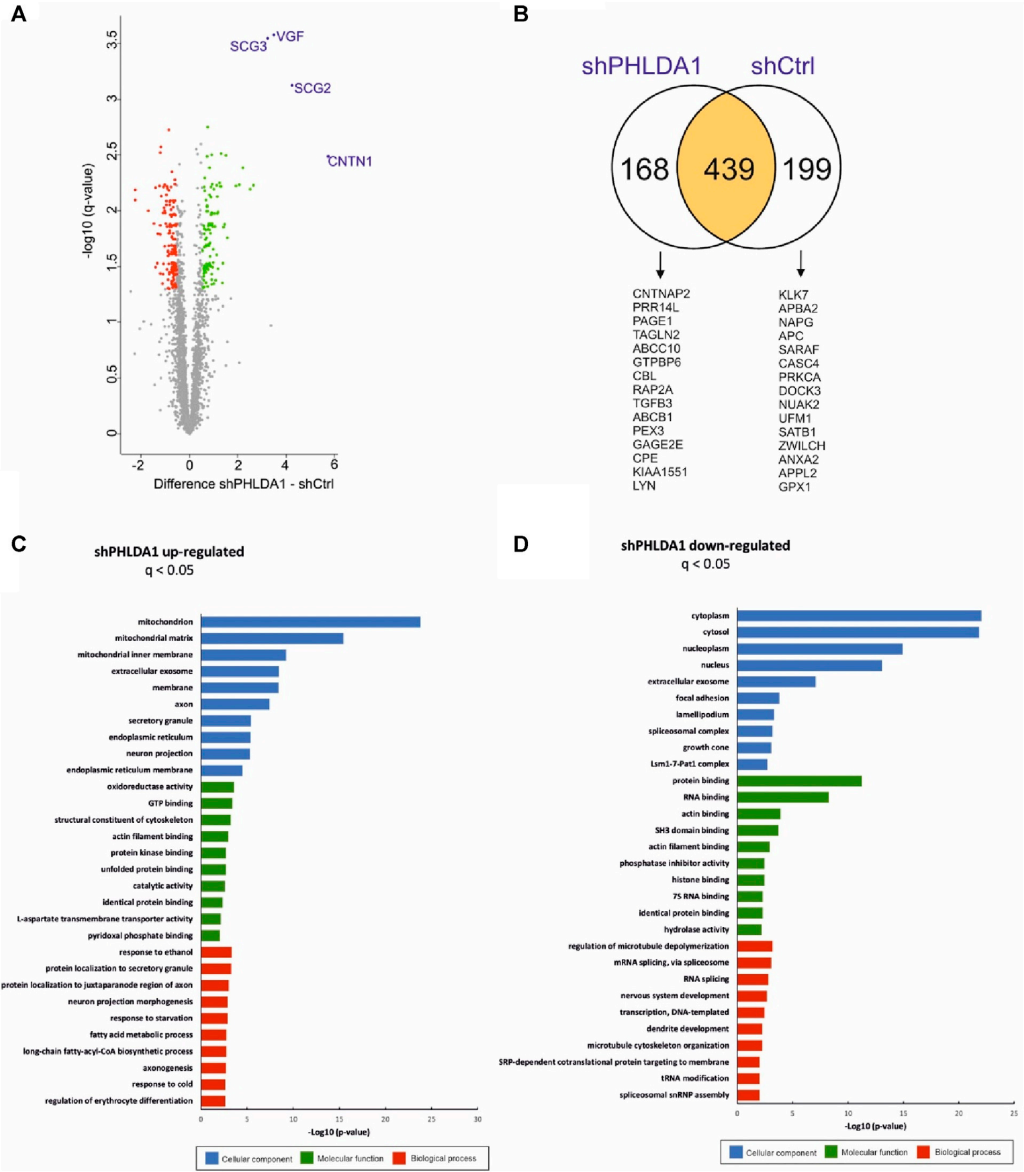
*PHLDA1* silencing leads to global upregulation of mitochondrial proteins. The volcano plot represents results from MS-based quantification of shPHLDA1 and shCtrl cells with five replicates in each group. Difference between averages of log2 protein intensities is plotted against -log10 q-value. Significantly altered proteins in shPHLDA1 that reached at least 1.45 – fold change (∓ 0.54 Difference threshold) and are below q value of 0.05 are colored green (upregulated) and red (downregulated) **(A)**. The Venn diagram displaying overlap between shPHLDA1 and shCtrl proteins on yellow background, shPHLDA1 and shCtrl specific proteins are presented on white backgrounds with top 15 proteins listed below, in order from the highest to the lowest signal difference between shPHLDA1 and shCtrl group. For a protein to be considered but present in only one group (shCtrl or shPHLDA1), it had to have at least three values in one group and one or zero in the other (see also Supplementary Table S3) **(B)**. Gene Ontology molecular function, biological process and cellular component terms enrichment analysis were prepared using the DAVID 6.8 platform for proteins statistically upregulated **(C)** and downregulated **(D)** in shPHLDA1 cells in comparison to shCtrl.

To resolve the biological roles of PHLDA1 in neuroblastoma, we performed quantitative proteomic analysis of *PHLDA1*-silenced vs. control IMR-32 cells. Overall, we detected around 3,500 proteins that exhibited at least 3 quantitative signals values in shPHLDA1 and/or shCtrl group (Supplementary Table S2). 439 proteins reached significance threshold after the multiple comparison correction (q < 0.05), out of which 250 exhibited at least 1.5-fold change between groups. 114 of these proteins were upregulated and 136 downregulated after *PHLDA1* inhibition (Figure 4A, Supplementary Table S2). 168 proteins were detected only in shPHLDA1 cells, and 199 proteins were specific only for shCtrl cells (Figure 4B, Supplementary Table S2). Proteins that were significantly altered and specific for one group were subjected to gene ontology annotation analysis using the DAVID 6.8 platform (Huang et al., 2009; Sherman et al., 2022). It appears that *PHLDA1* silencing causes the most pronounced changes in mitochondrion related proteome (Figure 4C), whereas proteins associated with cellular components of the cytoplasm are decreased in the cells (Figure 4D).

### 3.6 Low level of PHLDA1 is associated with adrenergic cell type of neuroblastoma

Recently it has been shown that neuroblastoma cells are composed of two, mesenchymal (MES) and adrenergic (ADRN), differentiation states (van Groningen et al., 2017). Our bioinformatic analyses of multiple datasets available on R2 revealed that the low level of *PHLDA1* gene expression associates with the adrenergic, more neuronal-directed state of neuroblastoma (Supplementary Figures S8A–D). Finally, we compared the set of genes coding for statistically upregulated and downregulated proteins, identified in our mass spectrometry analysis in *PHLDA1*-silenced vs. control cells, and previously described mesenchymal- and adrenergic-signature genes in neuroblastoma (van Groningen et al., 2017). Analysis revealed that the largest group of genes overlaps between the adrenergic and shPHLDA1 upregulated group (Supplementary Figures S8E).

### 3.7 *PHLDA1* silencing affects the phosphorylation of multiple proteins

To further explore the function of PHLDA1 we performed analysis of post-translational modifications of proteins in shPHLDA1 vs. shCtrl cells, using mass spectrometry. We can observe slight changes in ubiquitination levels of a few proteins, where only one, C1R, reached the significance threshold after the multiple comparison correction (Supplementary Table S3). Analysis of global phosphorylation changes revealed 156 peptides from 128 different proteins that had their phosphorylation status altered upon *PHLDA1* downregulation (Supplementary Figure S9A, Supplementary Table S4). The great majority (100) of the phosphorylated peptides exhibits downregulated phosphorylation status in shPHLDA1 vs. shCtrl cells whereas only 56 of peptides shows upregulation of phosphorylation pattern. Detected proteins were associated with multiple signaling pathways (Supplementary Figures S9B, S9C) and various kinases were predicted to potentially affect the phosphorylation status of detected proteins (Supplementary Figures S9D, S9E).

### 3.8 *PHLDA1* silencing augments EGF signaling pathway

**FIGURE 5:**
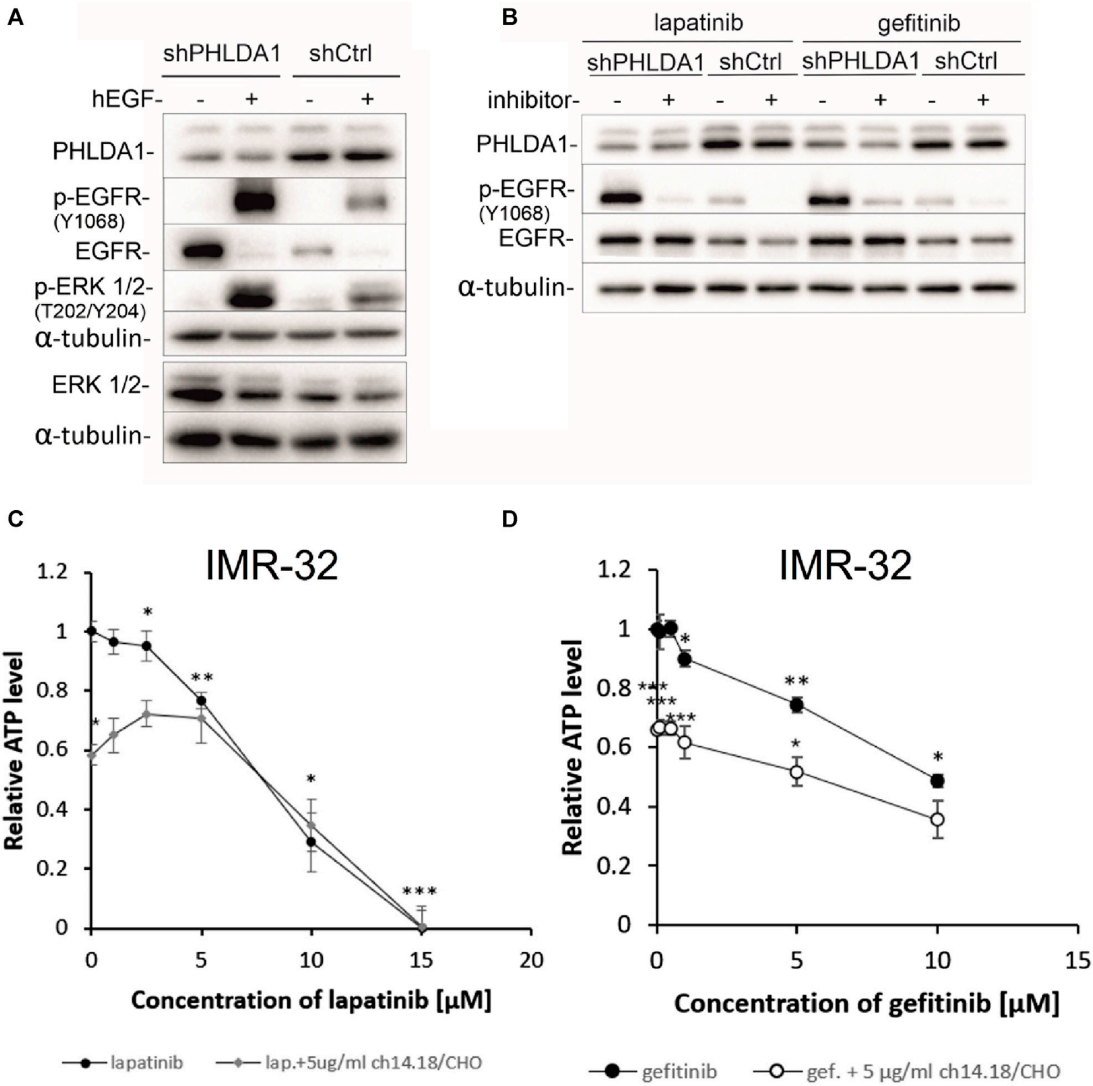
Inhibiting the EGFR signaling pathway decreases viability of neuroblastoma cells. shCtrl and shPHLDA1 IMR-32 cells were stimulated with hEGF, at concentration of 20 ng/mL for 15 min **(A)** or treated with EGFR inhibitors, lapatinib or gefitinib, at concentration of 5 μM for 72 h **(B)**. 0.1% BSA in PBS and DMSO were used as solvent controls for hEGF and inhibitors treatment, respectively. Then, cells were lysed, and Western blot analysis was performed using indicated antibodies. α-tubulin was used as a reference protein. shPHLDA1 – cells with silenced *PHLDA1*. shCtrl–mock cells, transfected with control plasmid. Results are shown as representative immunoblots of three independent experiments. IMR-32 cells were treated with lapatinib (1–15 μM) **(C)** or gefitinib (0.1–10 μM) **(D)**, and combinations of inhibitor and ch14.18/CHO antibody (5 μg/mL) for 72 h. Viability was assessed via ATP content measurements, in comparison to controls treated with diluents (DMSO for lapatinib/gefitinib and PBS for ch14.18/CHO, set as 1). Data are shown as means of three independent experiments ( ± SEM). Statistical significance of changes between different concentrations of inhibitors and diluent was determined by *t*-test (*p < 0.05, **p < 0.01, ***p < 0.001). Statistical significance between inhibitor alone and combination was determined by two-way ANOVA with the *post hoc* Tukey test (*p < 0.05, **p < 0.01, ***p < 0.001).

Using the phospho-RTK protein array in the preliminary studies, we discovered that phosphorylation of EGFR is activated, while phosphorylation of IGF-1R is inhibited upon *PHLDA1* downregulation in IMR-32 neuroblastoma cells transduced with a lentiviral *PHLDA1*-silencing vector as compared with control (Supplementary Figure S10). Thus, we extended the research to further investigate the role of EGFR and its connotation with PHLDA1 in human neuroblastoma. We compared the effect of stimulation with hEGF (20 ng/mL) and EGFR inhibitors treatment (lapatinib/gefitinib, 5 µM) on *PHLDA1*-silenced and control cells (Figures 5A,B). The results of Western blot imaging revealed that cells with silenced *PHLDA1* display the significantly higher EGFR phosphorylation level at tyrosine 1,068 (the main EGFR activating phosphorylation), what indicates a stronger stimulation of the EGF receptor pathway. However, the higher level of the phosphorylated form can be the result of the increased total EGFR level in shPHLDA1 cells, confirmed by mass spectrometry analysis (Supplementary Table S2) and Western blot (Figure 3B). Similarly, *PHLDA1*-silenced cells display enhanced activation of ERK1/2, which is one of the downstream targets of the EGF/EGFR signaling pathway (Ho et al., 2005).

The EGF signaling pathway overactivation is strongly involved in cancer progression and is often targeted during anti-cancer treatment (Ayati et al., 2020). To investigate whether inhibition of EGFR signaling might have beneficial effect for neuroblastoma patients we treated IMR-32 neuroblastoma cells with increasing doses of EGFR inhibitors: lapatinib (0.1–10 μM) and gefitinib (0.5–15 μM) alone and in combination with 5 μg/mL of ch14.18/CHO therapeutic antibody for 72 h and performed ATP measurements (Figures 5C,D). The observed cytotoxic effects were dose-dependent and statistically significant for both inhibitors, when compared to unstimulated control (*p < 0.05, **p < 0.01, ***p < 0.001). The obtained values of measured relative ATP levels were significantly lower for cells treated with gefitinib and antibodies (*p < 0.05, ***p < 0.001) than with gefitinib alone (Figure 5D). In case of lapatinib, combinatory treatment significantly decreased the relative ATP values in comparison to inhibitor alone only for the dose of 0.1 μM, (*p < 0.05) (Figure 5C). These observations are further supported by the comparison of calculated IC50 values for combination vs. inhibitor alone treatment. IC50 was lower for combination of gefitinib and ch14.18/CHO (IC50 = 5.38 ± 0.01) than for single gefitinib treatment (IC50 = 9.74 ± 0.03). However, there was no decrease of IC50 value of combinatory treatment than lapatinib treatment alone.

### 3.9 Impact of EGF inhibitors on *PHLDA1*-silenced IMR-32 cells

**FIGURE 6:**
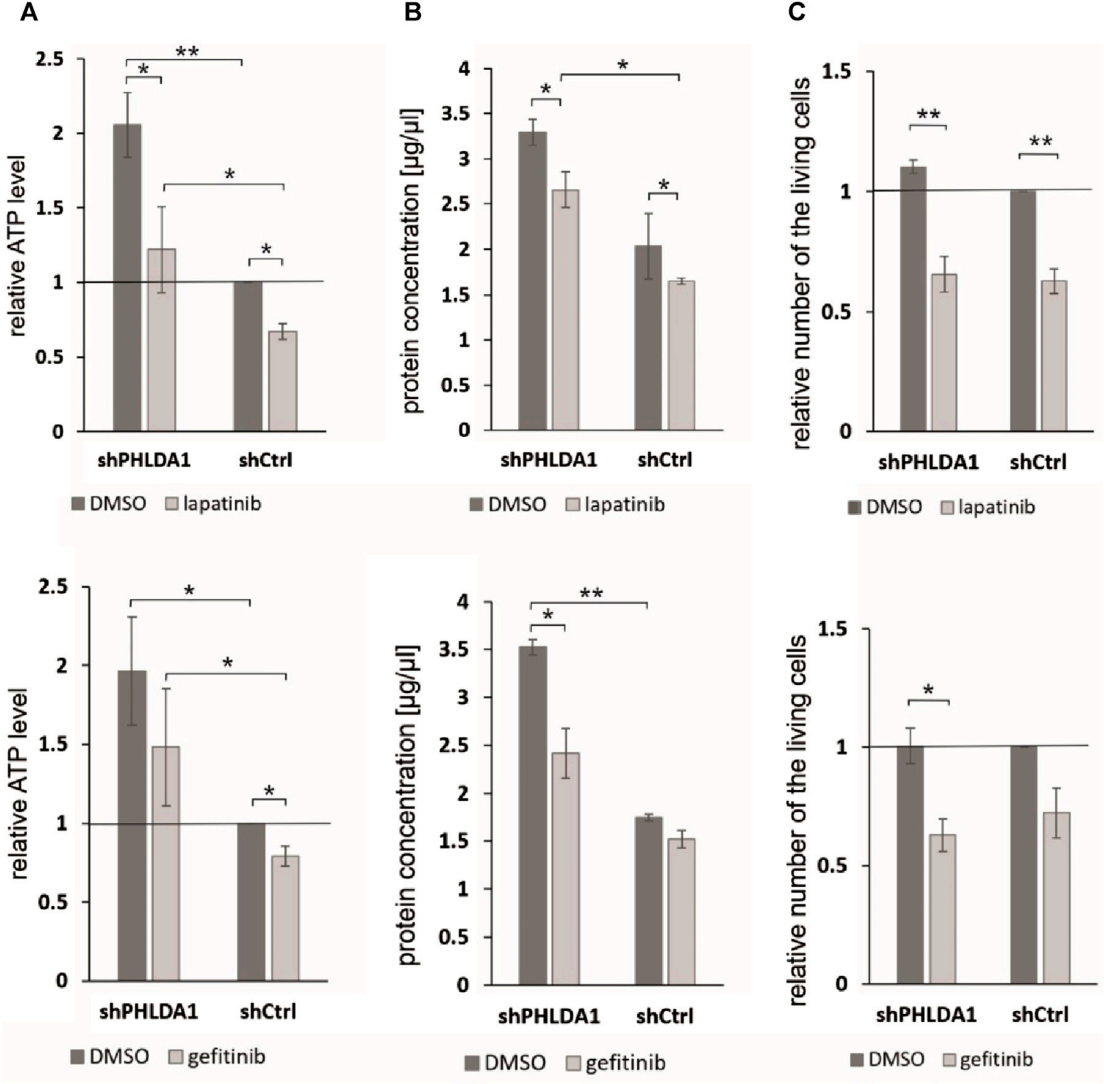
*PHLDA1* silencing sensitizes neuroblastoma cells to EGFR inhibitors treatment. *PHLDA1*-silenced (shPHLDA1) and control (shCtrl) cells were treated with 5 μM gefitinib/lapatinib or DMSO and seeded for 72 h. Relative ATP levels **(A)**, protein concentration in lysate **(B)** and the relative number of the living cells **(C)** were measured. The relative ATP levels and numbers of the living cells were compared to DMSO-treated shCtrl cells (set as 1). Protein concentration was measured via the BCA method in cell lysates isolated with RIPA buffer. Data are shown as a mean ( ± SEM) of at least three independent experiments. Statistical significance was determined by two-way ANOVA with *post hoc* Tukey test (*p < 0.05, **p < 0.01).

To investigate whether *PHLDA1* silencing affects the cytotoxicity caused by EGF inhibitors, we treated the modified IMR-32 cells with lapatinib or gefitinib and measured ATP levels, protein concentrations and viability via the trypan blue test. We observed that the relative ATP level is decreased in shPHLDA1 and shCtrl cells treated with both inhibitors: lapatinib and gefitinib (Figure 6A). Statistically significant decrease was observed for both shPHLDA1 (*p* < 0.05) and shCtrl (*p* < 0.05) in lapatinib-treated cells, while only in shCtrl (*p* < 0.05) in gefitinib-treated cells. Moreover, the ATP test pointed that cells with *PHLDA1* downregulation manifest nearly 2-fold increase in ATP level than shCtrl cells (*p* < 0.05, *p* < 0.01), which corresponds to upregulation of mitochondria-related proteins discovered via mass spectrometry analysis and increased mitochondrial membrane potential, previously shown by our group (Durbas et al., 2016). The protein concentration significantly decreases in shPHLDA1 cells treated with both EGFR inhibitors (Figure 6B) and in shCtrl cells treated with lapatinib (*p* < 0.05). Relative number of the living cells heavily decreases in shPHLDA1 and shCtrl cells treated with lapatinib (*p* < 0.01) and shPHLDA1 cells treated with gefitinib. On the contrary, the number of the dead cells exhibits growing tendency in inhibitors-treated cells (Supplementary Figure S13A). Both inhibitors treatments significantly decrease total cell number of *PHLDA1*-silenced cells. Taken together, our data imply that *PHLDA1* silencing enhances the EGF receptor signaling pathway, but the influence is unidirectional since neither EGFR activation, nor inhibition does not generate changes in the PHLDA1 level in shCtrl cells (Figures 5A,B) and IMR-32 neuroblastoma cells (Supplementary Figures S11, S12).

## 4 Discussion

Our transcriptomic and proteomic analyses aimed at elucidation of the role of PHLDA1 in neuroblastoma, as its role in different cancers remains not yet fully elucidated. Analysis of the publicly available dataset revealed that the level of mRNA of *PHLDA1* is positively correlated with survival of neuroblastoma patients with *MYCN*-amplification, but no significant correlation was found for the larger group of patients with *MYCN*-non-amplified status in the analyzed dataset. This suggests that patients with *MYCN*-amplification might benefit the most from anti-GD2 treatment which significantly activates *PHLDA1* expression, at least in IMR-32 neuroblastoma cell line (Horwacik et al., 2015). Nevertheless, more data is needed to establish whether *PHLDA1* is activated upon anti-GD2 treatment in neuroblastoma patients. The correlation of the *PHLDA1* expression and the *MYCN*-amplification status might be associated with aurora A kinase activity, as we have previously shown that *PHLDA1* silencing increases the aurora A level (Durbas et al., 2016). Additionally, the kinase is the main factor responsible for MYCN stability in neuroblastoma (Otto et al., 2009). Thus, it would be of interest to establish, if the higher expression of *PHLDA1* in NB patients decreases aurora A levels and consequently destabilizes MYCN.

In order to investigate how the ch14.18/CHO-treatment affects PHLDA1 protein interactors in neuroblastoma cells, we compared potential PHLDA1 binding proteins in the control- and the ch14.18/CHO antibody-treated IMR-32 cells by co-immunoprecipitation, using anti-PHLDA1 antibodies followed by mass spectrometry. This proteomic approach enabled confirmation of previously described- and presentation of new PHLDA1-binding proteins. PHLDA1 binding partners, 103 out of 111, exist in the experimentally confirmed complexes (Figure 1A). They belong to different processes (Supplementary Figure S3), such as signaling of Rho GTPases, which are associated with the phosphatidylinositol signaling pathway, where PHLDA1 was also described to be involved in (Chen et al., 2018).

We would like to point out that two novel PHLDA1-binding proteins, DCAF7 and AUTS2, gained our attention in our model, as treatment of IMR-32 cells with ch14.18/CHO affected levels of both proteins. Importantly, their binding to PHLDA1 protein was confirmed by us by mass spectrometry and co-immunoprecipitation, although a very low expression of endogenous AUTS2 protein in IMR-32 cells was observed. Moreover, a chaperone CCT3 was identified among the constitutive PHLDA1 binding candidates (Figure 1A) and it was already described to exist in physical complex with DCAF7 protein (also known as WDR68), which was also described to interact with AUTS2 (Figure 1A) (Miyata et al., 2014; Wang et al., 2018). Interestingly, we also stress the importance of AUTS2 in neuroblastoma by presenting its mRNA positive correlation with survival of neuroblastoma patients (Supplementary Figures S6C, D). DCAF7 and AUTS2 belong to the Polycomb Repressive Complex 1 (PRC1) and together they are involved in the transcriptional activation of neurodevelopmental genes (Wang et al., 2018). This may suggest that treating neuroblastoma cells with anti-GD2 antibodies may lead to a reduction in the differentiation process. Here we show that PHLDA1 might inhibit differentiation, because *PHLDA1-*silenced IMR-32 cells exhibit differentiation-like phenotype accompanied by profound increase of the mitochondrial proteome. Furthermore, our bioinformatic analysis indicates that PHLDA1 downregulation might weaken the mesenchymal and enhance adrenergic state of neuroblastoma cells (Supplementary Figure S8). The mechanism by which PHLDA1 is involved in regulation of neuroblastoma differentiation might relay on its direct interaction with DCAF7/AUTS2 complex, what might lead to alteration in the AUTS2 isoforms balance observed in this study. AUTS2 manifests a different expression pattern in IMR-32 cells with *PHLDA1* silencing - the level of the shorter AUTS2 isoform (around 100 kDa) decreases, while the level of the longer form (around 115 kDa) rises (Figure 3B). Different AUTS2 isoforms patterns were previously described repeatedly (Hori et al., 2014; Monderer-Rothkoff et al., 2021). Hori et al. suggested that deletion of one of AUTS2 isoforms is leading to a potentially compensatory increase of another AUTS2 isoform (Hori et al., 2014), what is also observed after *PHLDA1* silencing in our study (Figure 3B). Furthermore, mass spectrometry analysis of PHLDA1-co-immunoprecipitated proteins did not reveal any other members of the PRC1 other than DCAF7 and AUTS2 (Supplementary Table S1). Thus, it is also possible that PHLDA1 interacts with the DCAF7/AUTS2 complex that acts independently of the PRC1 complex. Recent study suggests that the DCAF7/AUTS2 complex can indeed promote neuronal differentiation through inhibition of BMP signaling, independently of the activity of the PRC1 complex (Geng et al., 2022). Furthermore, to analyze the biological roles of PHLDA1 in neuroblastoma, we performed quantitative proteomic analysis of *PHLDA1*-silenced vs. control IMR-32 cells and found that *PHLDA1* silencing causes the most spectacular increase in biological functions of mitochondria (Figure 4C), whereas proteins associated with cellular components of the cytoplasm are decreased in the cells (Figure 4D). Increase of mitochondrial biomass is a well-known feature of neuronal differentiation due to the higher need for ATP, necessary for neurite outgrowth and synaptic functions (Cheng et al., 2010).

The anti-GD2 immunotherapy was proven to significantly increase survival of neuroblastoma patients, but due to relatively high rate of relapse there is still high need for therapy improvement (DuBois et al., 2022). *PHLDA1* is the most upregulated gene in IMR-32 cells treated with the anti-GD2 14G2a antibody. Our previous data indicates that it probably has pro-apoptotic role in IMR-32 cells, but results presented here, with application of anti-GD2 ch14.18/CHO antibodies, indicate that at the same time PHLDA1 might inhibit differentiation of neuroblastoma cells. Our proteomic data from *PHLDA1-*silenced cells showed multiple proteins that are regulated by PHLDA1. We propose that our mass spectrometry data might be used to select new potential therapeutic targets in neuroblastoma. Our analysis revealed that in shPHLDA1-up-regulated group of proteins there is similar number of proteins which expression level is negatively and positively correlated with survival of patients, however in shPHLDA1 downregulated group, the number of proteins which expression negatively corelates with survival of patients is around 50% higher than the number of proteins linked to positive correlation with survival (Supplementary Figure S14A). Potentially, proteins that are downregulated in shPHLDA1 cells might be upregulated after ch14.18/CHO treatment and therefore they might be potential new targets that can be used in combination with ch14.18/CHO therapy if their expression negatively correlates with survival of neuroblastoma patients. We propose several such potential new targets (Supplementary Figures S14C–F).

Finally, we have investigated the molecular mechanism by which PHLDA1 upregulates the EGFR protein level, which might be associated with enhanced EGFR receptor stability, due to direct interaction of EGFR receptor with the CaMKII kinase. It has been shown that CaMKII regulates both EGFR kinase activity and the rate of its endocytosis (Sánchez-González et al., 2010). CaMKII was identified in this study as potential PHLDA1-binding partner and *PHLDA1* silencing leads to downregulation of the CaMKII kinase level what might decrease rate of EGFR endocytosis and lysosomal degradation. Observed differences between impact of lapatinib and gefitinib on IMR-32 cells might result from different specificity spectrum of these two inhibitors, as gefitinib is EGFR (HER1)-specific and lapatinib also inhibits other members of receptor tyrosine kinases (Gilmer et al., 2008; Segovia-Mendoza et al., 2015). Additionally, combined treatment with ch14.18/CHO does not improve the cytotoxicity of lapatinib (Figure 5C), but enhances the cytotoxic effect of gefitinib, especially when treated with low doses of the inhibitor (Figure 5D). Presented data indicated that *PHLDA1* silencing alone causes significant upregulation of ATP content and concentration of isolated proteins in samples. It is most likely not the result of an increased proliferation rate of shPHLDA1 cells, as proved by cell counting and previously showed an upregulated percentage of *PHLDA1*-silenced cells in the G0/G1 phase of the cell cycle (Durbas et al., 2016). Furthermore, almost all measured parameters (except the number of dead cells) show stronger impact of EGFR inhibitors on *PHLDA1*-silenced cells than on shCtrl cells. This phenomenon might result from increased expression of EGFR in *PHLDA1*-silenced cells and consequently a higher dependence of these cells on the EGFR signaling pathway. Taken together, our data indicate that silencing of *PHLDA1* sensitizes IMR-32 neuroblastoma cells to EGFR inhibitors treatment.

Additionally, our combined analysis of PHLDA1 binding candidates and shPHLDA1-altered proteome pointed to the involvement of PHLDA1 in the axon guidance pathway (Supplementary Figure S15). Axon guidance molecules are considered as tumorigenic and anti-cancer targets (Chédotal et al., 2005; Rached et al., 2016; Zaatiti et al., 2018), however, in neuroblastoma, genes involved in axon guidance are often mutated (Li et al., 2017). More detailed analysis revealed that among several signaling pathways affected by PHLDA1 within “axon guidance pathway”, “L1CAM interactions” and “signaling by ROBO receptor” are the most significantly enriched (Supplementary Figure S15B). Regulation of ROBOs receptors and Rho GTPases were also detected among pathways affected by PHLDA1-binding candidates in control and ch14.18/CHO-treated cells (Supplementary Figure S3) what indicates the involvement of these pathways in PHLDA1-dependent ch14.18/CHO response of neuroblastoma.

To summarize our results, we can conclude that the presented experimental data broaden our knowledge on direct cytotoxicity of the ch14.18/CHO mAb on IMR-32 cells, its effects on changes of cellular proteome, extend our understanding of a role of PHLDA1 in signaling pathways regulating neuroblastoma cell fate in the studied model, and propose new protein candidates relevant to neuroblastoma biology for further studies.

## 5 Scope statement

Neuroblastoma is the most common extracranial pediatric solid tumor in which GD2 ganglioside is a marker in diagnosis and a target in neuroblastoma immunotherapy with GD2 ganglioside-binding antibodies. We have used human neuroblastoma cells as a research model to investigate the direct cytotoxicity phenomenon induced upon binding of the clinically approved anti-GD2 ganglioside ch14.18/CHO antibody. Targeting the ganglioside with the therapeutic antibodies affects several pivotal signaling routes that drive or influence the malignant phenotype of the cells. One of our research goals is to deepen knowledge on mechanism of signaling involving gangliosides that leads to tumor cell death without involvement of the immune system. Previously performed gene expression profiling helped us to identify the *PHLDA1* (pleckstrin homology-like domain family A member 1) gene as the most upregulated gene in human IMR-32 neuroblastoma cells treated with the mouse 14G2a monoclonal antibody. In the present study, cell culture experiments combined with proteomic analyses (mass spectrometry, co-immunoprecipitation, Western blotting) were applied to better characterize a role of PHLDA1 protein in the response of IMR-32 human neuroblastoma cells to chimeric ch14.18/CHO antibody. For example, our analyses suggest that PHLDA1 directly interacts with the DCAF7/AUTS2 complex, a key component of neuronal differentiation *in vitro*.

## Data Availability

The datasets presented in this study can be found in online repositories. The names of the repository/repositories and accession number(s) can be found in the article/Supplementary Material. Data are available via ProteomeXchange (https://www.ebi.ac.uk/pride/), with the identifier PXD044319.

## Abbreviations

ADRN, adrenergic; AUTS2, autism susceptibility gene 2 protein; BMP, bone morphogenetic protein 1; CaMKII, calcium/calmodulin-dependent protein kinase type II; CCT3, T-complex protein 1 subunit gamma; CDKN1A, cyclin dependent kinase inhibitor 1A; C1R, complement C1r; DCAF7, DDB1- and CUL4-associated factor 7; EGFR, epidermal growth factor receptor; GD2, disialoganglioside GD2; IGF-1R, insulin-like growth factor 1 receptor; L1CAM, neural cell adhesion molecule L1; MES, mesenchymal; MYCN, N-myc proto-oncogene protein; NB, neuroblastoma; PAR-4, prostate apoptosis response-4; PHLDA1, pleckstrin homology-like domain family A member 1; PRC1, polycomb repressive complex 1; ROBO, roundabout family receptors; SCG2, secretogranin-2; TRKB, neurotrophic receptor tyrosine kinase 2.
