# Supplementary material for: Silencing of the PHLDA1 leads to global proteome changes and differentiation pathways of human neuroblastoma cells
Source: Front Pharmacol. 2024 Mar 1;15:1351536. doi: 10.3389/fphar.2024.1351536 (PMC10941682; doi:10.3389/fphar.2024.1351536)
Supplement: Supplementary file 8 [file Table8.pdf]

Table S 8. Antibodies used in the study.

Antigen	Host species	Dilution	Vendor	Catalog no.
AUTS2	Mouse	1:1000	Abcam	ab243036
CaMKII (pan)	Rabbit	1:1000	Cell Signaling Technology	4436
DCAF7	Rabbit	1:5000	Abcam	ab138490
EGFR	Rabbit	1:1000	Cell Signaling Technology	4267
p-EGFR Y1068	Rabbit	1:1000	Cell Signaling Technology	3777
ERK 1/2	Rabbit	1:2000	Cell Signaling Technology	4695
p-ERK 1/2 T202/Y204	Rabbit	1:2000	Cell Signaling Technology	4370
GAPDH	Mouse	1:20.000	Sigma-Aldrich	G8795
IGF-1R	Rabbit	1:1000	Cell Signaling Technology	9750
mTOR	Rabbit	1:1000	Cell Signaling Technology	2983
p-mTOR S2448	Rabbit	1:1000	Cell Signaling Technology	5536
Nestin	Mouse	1:300	Santa Cruz	Sc-23927

Nanog	Rabbit	1:500	Cell Signaling Technology	4903
PHLDA1	Mouse	1:1000	Santa Cruz	Sc-23866
SCG2	Rabbit	1:1000	Abcam	ab2126935
Ubiquityl-H2A K119	Rabbit	1:1000	Cell Signaling Technology	8240
α-tubulin	Rabbit	1:4000	Cell Signaling Technology	2125
β-actin	Mouse	1:4000	Sigma-Aldrich	A1978
Secondary anti-rabbit IgG	Goat	1:2000-1:4000	Cell Signaling Technology	7074
Secondary anti-mouse IgG	Horse	1:3000	Cell Signaling Technology	7076
Secondary anti-human (Fc specific)	Goat	1:90.000	Sigma-Aldrich	A0170
