# Supplementary material for: Silencing of the PHLDA1 leads to global proteome changes and differentiation pathways of human neuroblastoma cells
Source: Front Pharmacol. 2024 Mar 1;15:1351536. doi: 10.3389/fphar.2024.1351536 (PMC10941682; doi:10.3389/fphar.2024.1351536)
Supplement: Supplementary file 9 [file DataSheet1.docx]

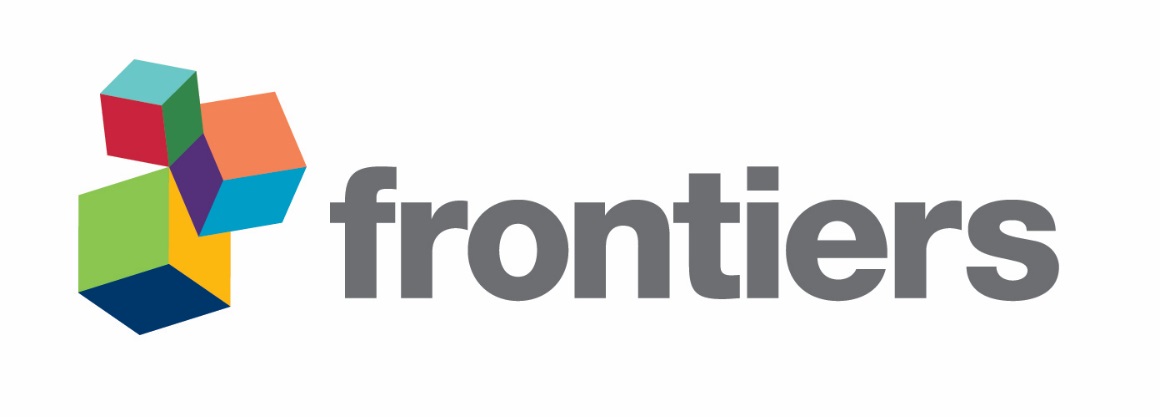


Supplementary Material

# Supplementary Figures and Tables

## Supplementary Figures


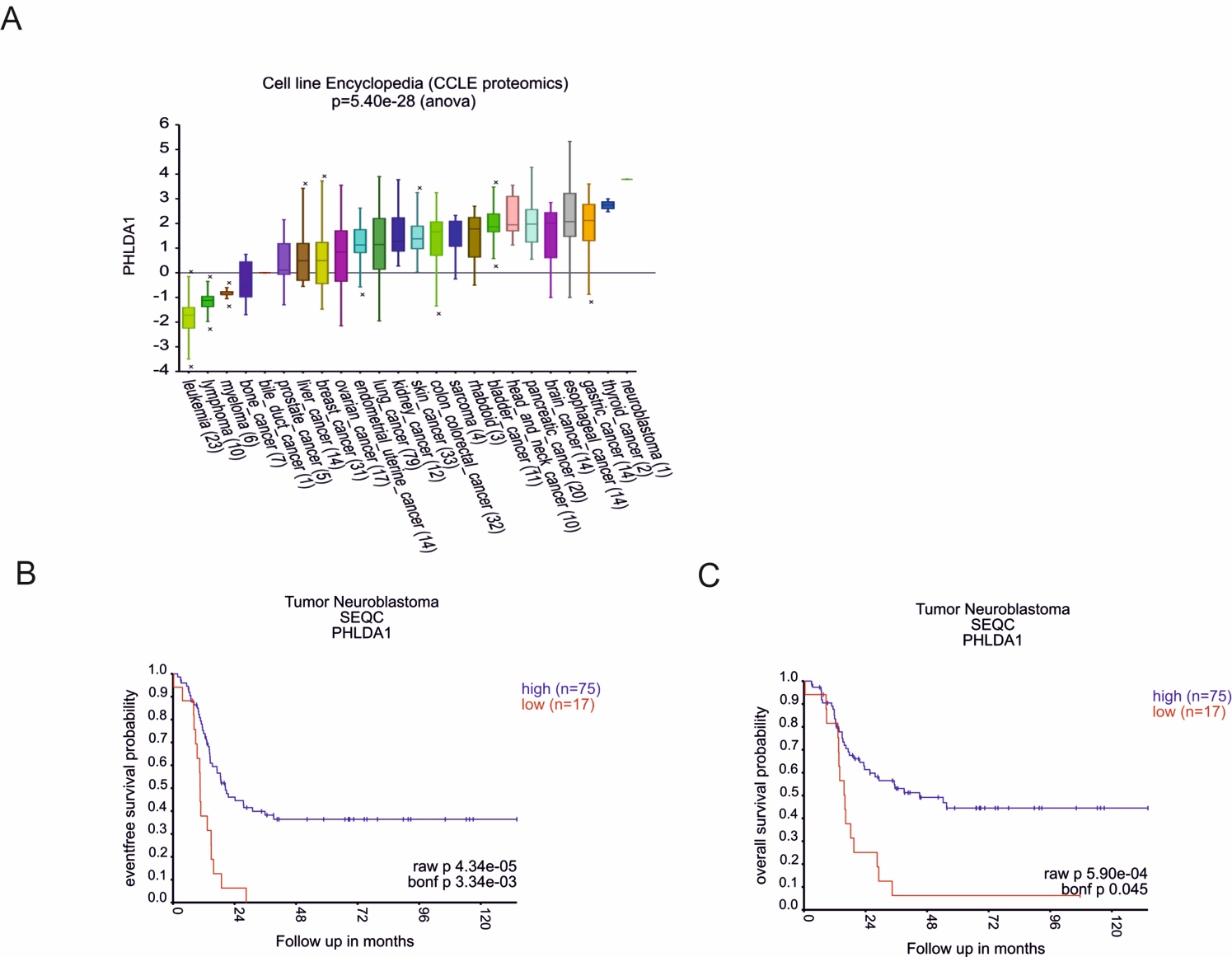
**Supplementary Figure 1. *PHLDA1* expression positively correlates with survival of patients with *MYCN*-amplified neuroblastoma.** Bioinformatic analysis was performed by using tools implemented in R2 (http://r2.amc.nl, <http://r2platform.com>). PHLDA1 protein level was compared between cell lines from 24 cancer types of Cancer Cell Line Encyclopedia (CCLE-Broad) proteomics dataset of 378 samples (A). Kaplan-Meier graphs of correlations between *PHLDA1* mRNA level and event-free (B) and overall (C) survival of neuroblastoma patients from SEQC RNA sequencing dataset (GSE62564), from the subset of 92 samples classified as *MYCN*-amplified.


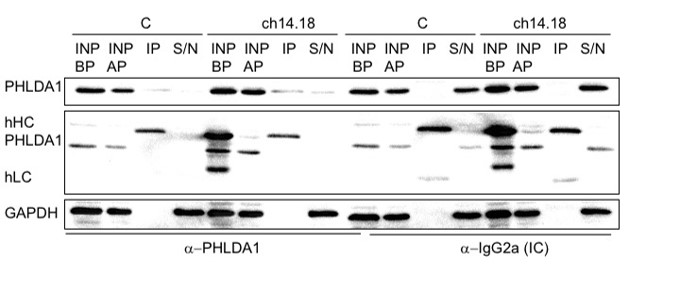


**Supplementary Figure 2. Representative immunoblots after PHLDA1 immunoprecipitation.** C – control IMR-32 cells treated with PBS; ch14.18 – ch14.18/CHO-treated IMR-32 cells; α-IgG2a – isotypic control (IC); hHC – human heavy chains of ch14.18/CHO Abs; hLC – human light chains of ch14.18/CHO Abs; INP – input; IP – immunoprecipitate; S/N – supernatant; BP – before preclearing; AP – after preclearing.


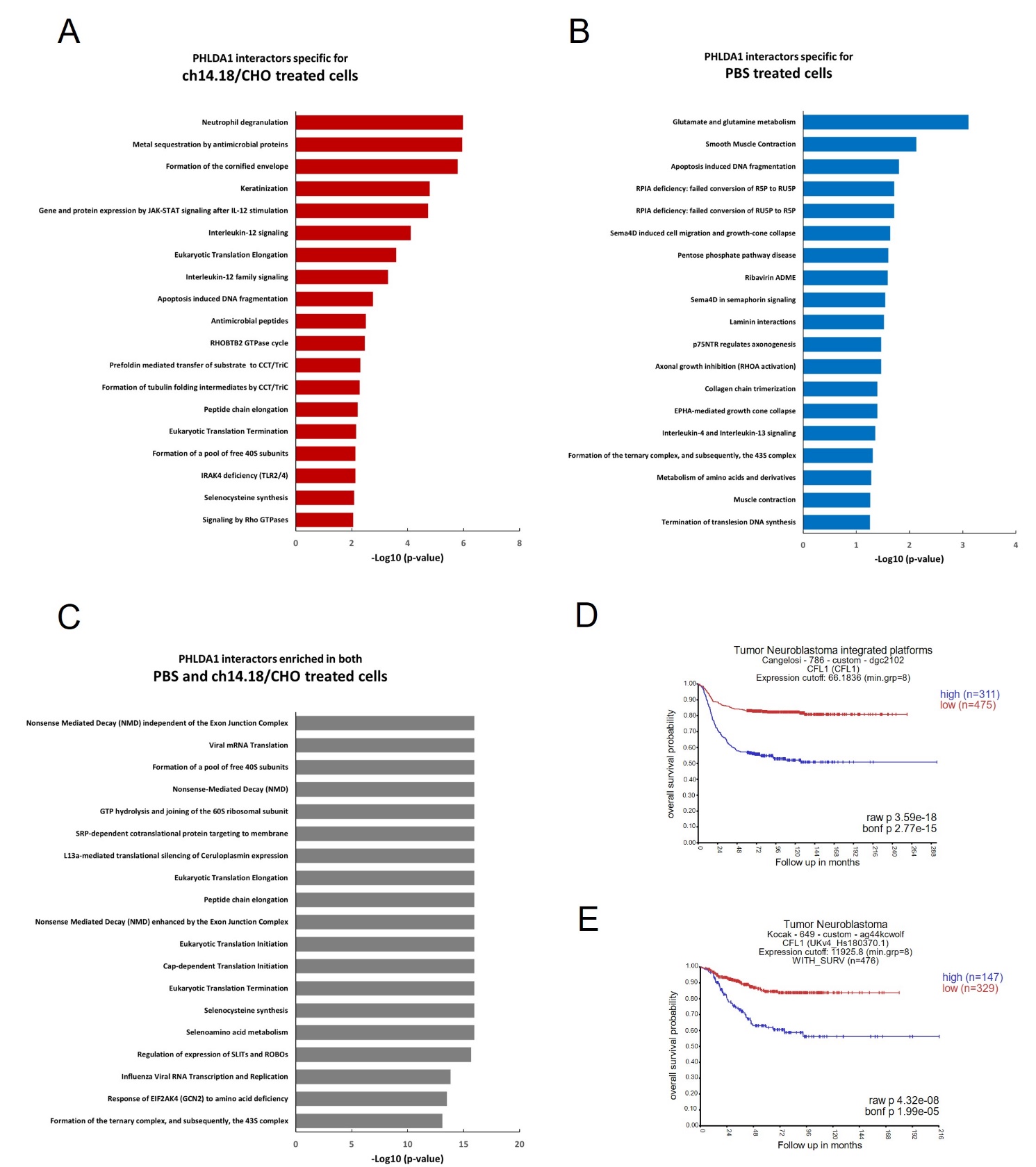


**Supplementary Figure 3. PHLDA1 binding candidates participate in various cellular pathways.** Potential PHLDA1 interactors specific for ch14.18/CHO- (A), PBS-treated (B) or enriched in both PBS ch14.18/CHO groups (C) detected in mass spectrometry analysis were subjected to pathway enrichment analysis tool, Reactome. Kaplan-Meier graphs of correlations between the *CFL1* mRNA level and overall survival of neuroblastoma patients from gene expression Cangelosi-786 (D), Kocak-649 (GSE45547) (E) datasets available on R2 (http://r2.amc.nl, <http://r2platform.com>).


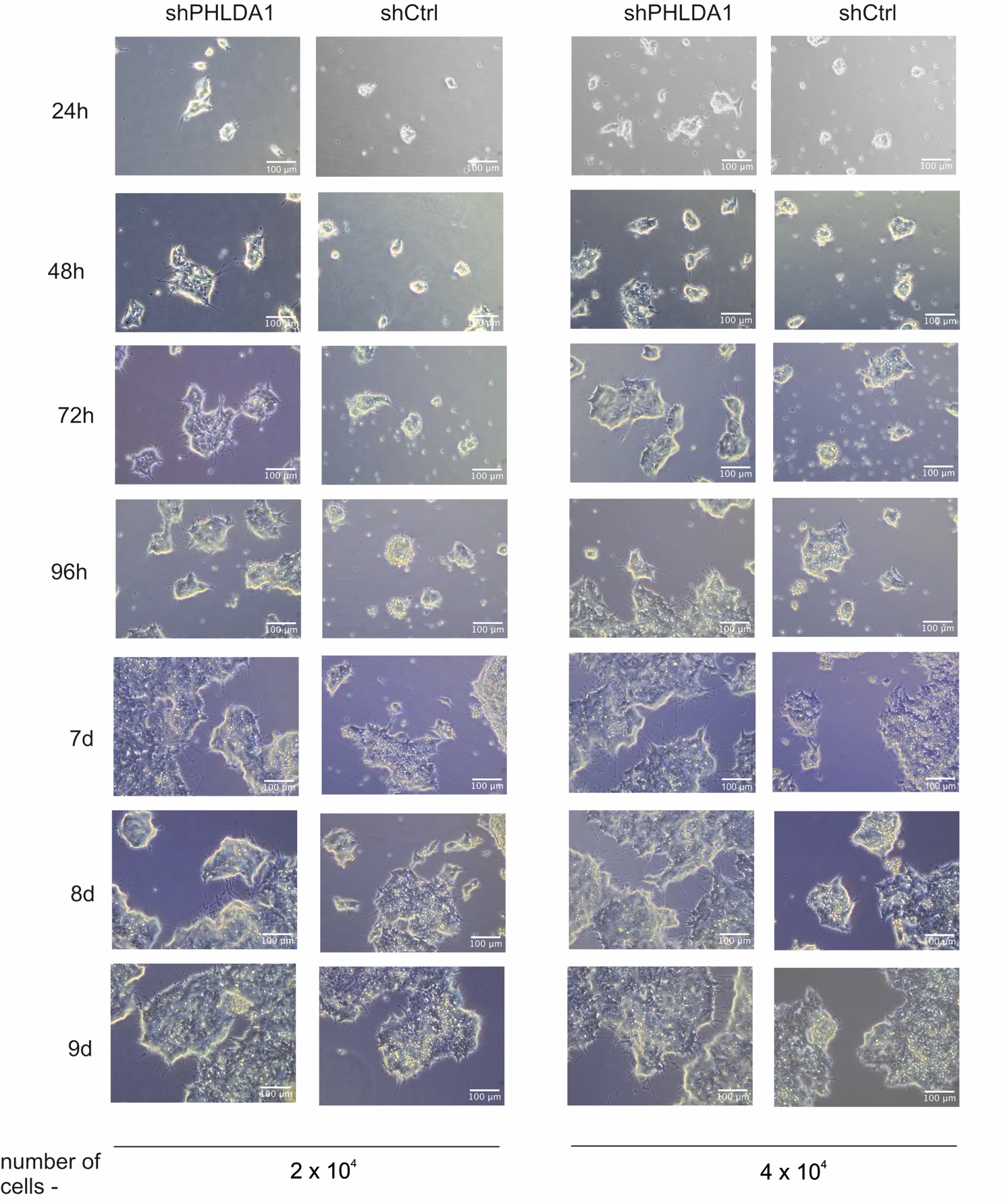


**Supplementary Figure 4. Microscopic observations of *PHLDA1*-silenced and control cells.** Microscopic images were captured at indicated time points between 24 h and 9 days (d) from seeding of 2 x 10^4^ (A) and 4 x 10^4^ (B) cells. shPHLDA1 – IMR-32 cells transfected with plasmid with shRNA against *PHLDA1*, shCtrl – IMR-32 cells transfected with control plasmid.


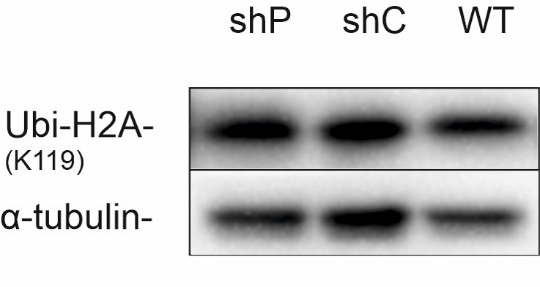


**Supplementary Figure 5. *PHLDA1*** **silencing has no impact on the level of ubiquitylated histone H2A at lysine 119**. Cells were seeded for 48 h, lysed and western blot analysis was performed using indicated antibodies. α-tubulin was used as reference protein. Results are shown as representative blots of three independent experiments. shP – IMR-32 cells transfected with plasmid with shRNA against *PHLDA1*, shC – IMR-32 cells transfected with control plasmid, WT – IMR-32 cells non-transfected with plasmid. Ubi-H2A -Ubiquitylated-Histone H2A.


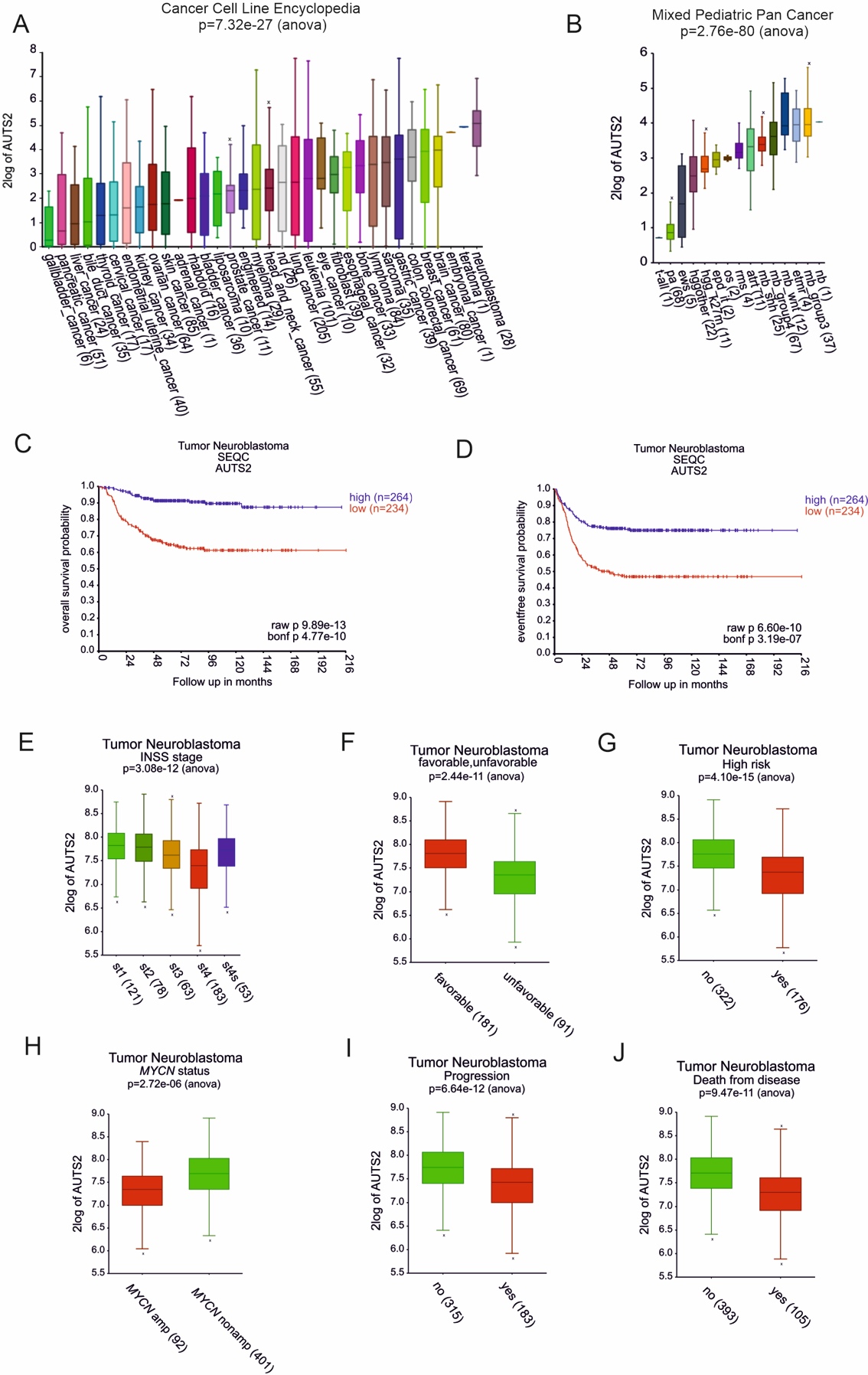


**Supplementary Figure 6. High *AUTS2* expression correlates with increased survival of neuroblastoma patients.** Bioinformatic analysis was performed by using tools implemented in R2 (http://r2.amc.nl, <http://r2platform.com>). *AUTS2* mRNA level was compared between cell lines from 34 cancer types of Cancer Cell line Encyclopedia (CCLE-Broad) dataset of 1389 samples (A) and between 15 tumors from Mixed Pediatric Pan Cancer dataset (B). *AUTS2* (NM_015570) mRNA expression level from Tumor Neuroblastoma SEQC mRNA microarray dataset (GSE62564) of 498 samples was correlated with overall (C) and event-free (D) survival of neuroblastoma patients, then compared between neuroblastoma INSS stages (E), favorable vs unfavorable status (F), high vs low risk (G), *MYCN* amplified vs non-amplified (H), disease with/without progression (I), death vs no death from the disease (J).


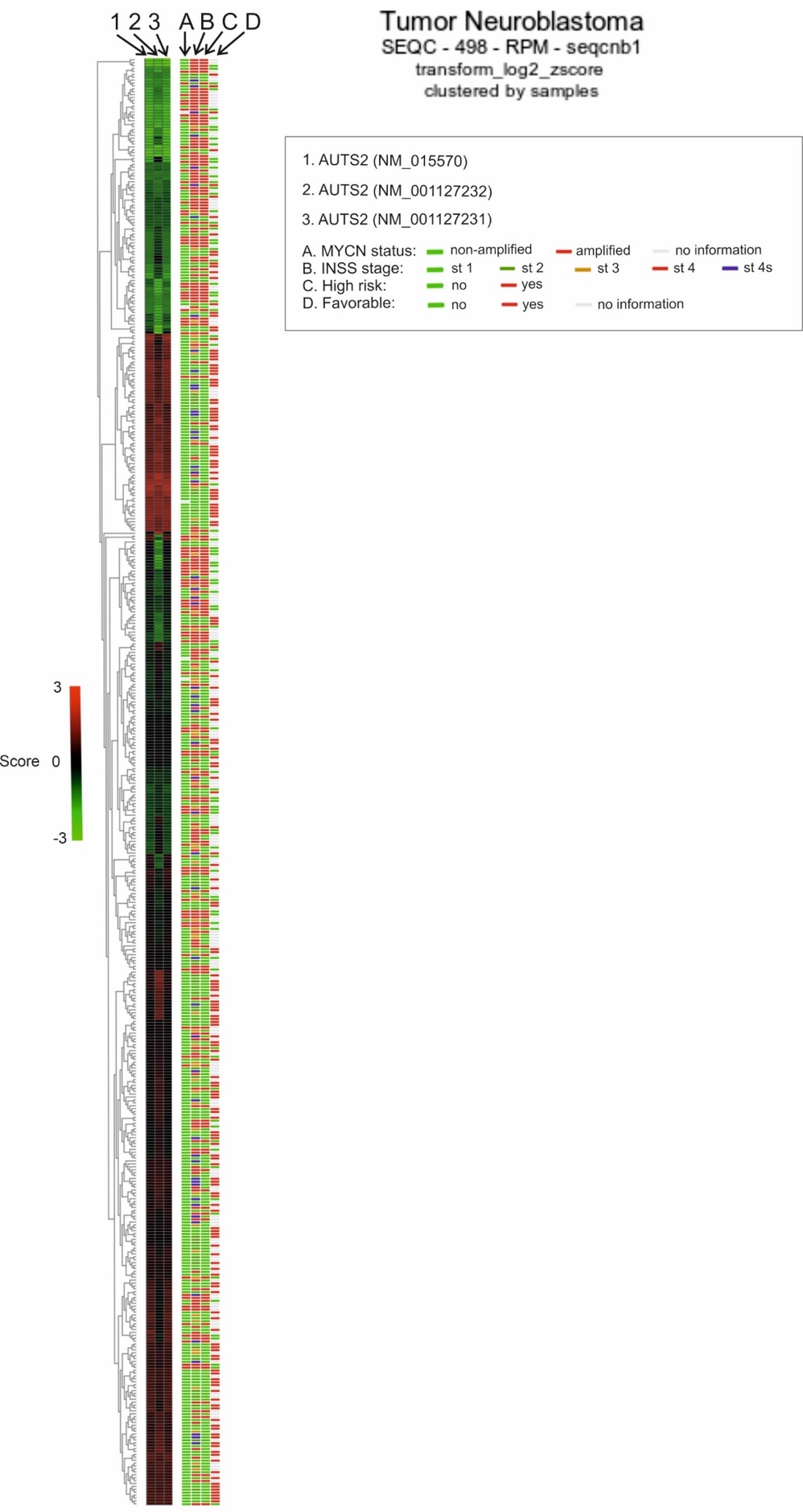


**Supplementary Figure 7. *AUTS2* isoforms expression exhibits similar correlation with the features of neuroblastoma tumours.** Bioinformatic analysis was performed by using tools implemented in R2 (http://r2.amc.nl, <http://r2platform.com>). Three *AUTS2* isoforms (NM_015570, NM_001127232, NM_001127231) expression levels were corelated with indicated features of neuroblastoma tumours from SEQC RNA sequencing dataset (GSE62564) of 498 samples ordered by clustering. Logarithmic transformation of “z” score was used to generate a heatmap.


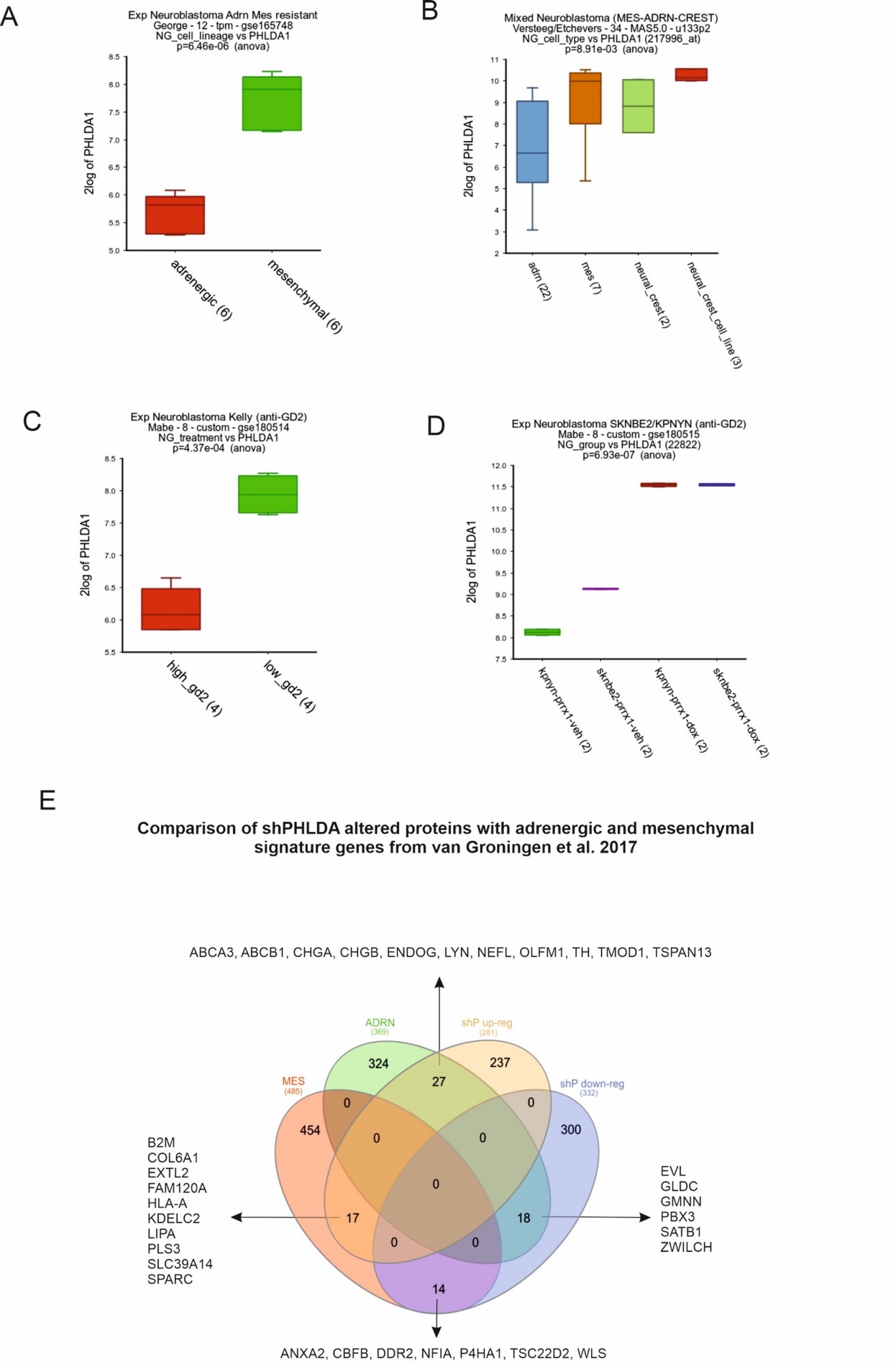


**Supplementary Figure 8. The low level of PHLDA1 is associated with the adrenergic cell type of neuroblastoma.** Bioinformatic analyses were performed by using tools implemented in R2 (http://r2.amc.nl, http://r2platform.com) of George-GSE165748 (A) and Versteeg/Etchevers-34 (B), Mabe-GSE180514 (C), Mabe-GSE180515 (D) mRNA datasets. Veh-vehicle, Dox-doxycycline. The Venn diagram shows overlapping genes coding for statistically up-regulated (shP up-reg) and down-regulated (shP down-reg) proteins, identified in mass spectrometry analysis of *PHLDA1*-silencing vs control cells, and previously described mesenchymal (MES) and adrenergic (ADRN) signature genes in neuroblastoma [57]. Subsets of overlapping genes that were specific for shPHLDA1 or shCtrl group were listed (E).


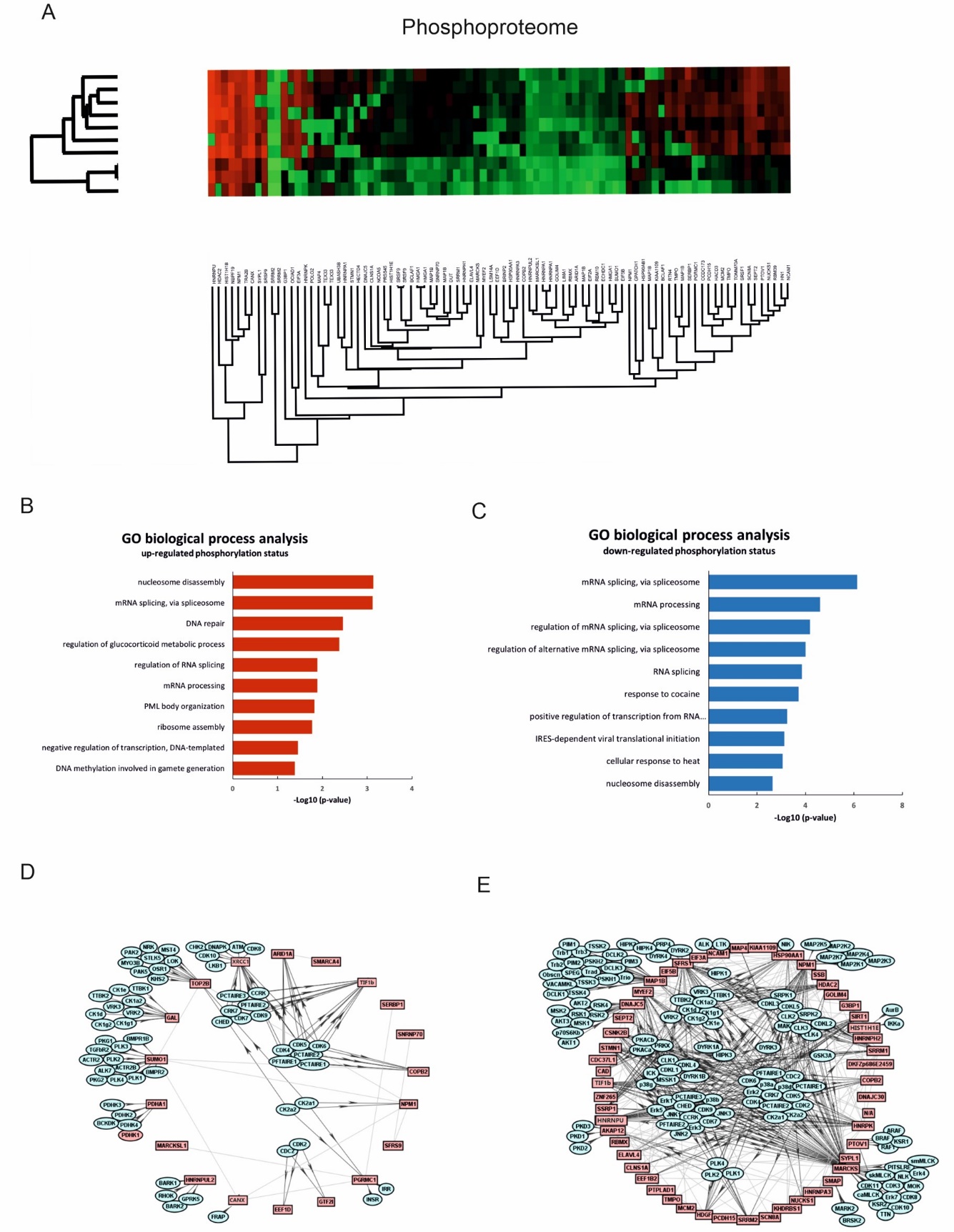


**Supplementary Figure 9. *PHLDA1* silencing alters phosphorylation of proteins related to nucleosome disassemble and mRNA splicing.** The heatmap represents the difference of phosphorylation status between shPHLDA1 and shCtrl cells of all proteins identified from total protein extracts of five independent experiments, ordered by clustering. (A). Gene Ontology analysis of biological processes was prepared using the DAVID 6.8 platform for all proteins with up-regulated (B) and down-regulated (C) phosphorylation status in shPHLDA1 cells in comparison to shCtrl. Prediction of site-specific kinase-substrate relations from phosphoproteome data was performed using the iGPS 1.0 software, separately for identified proteins with up-regulated (D) and down-regulated (E) phosphorylation status. Predicted kinases are colored light green and analyzed substrates are colored light red.


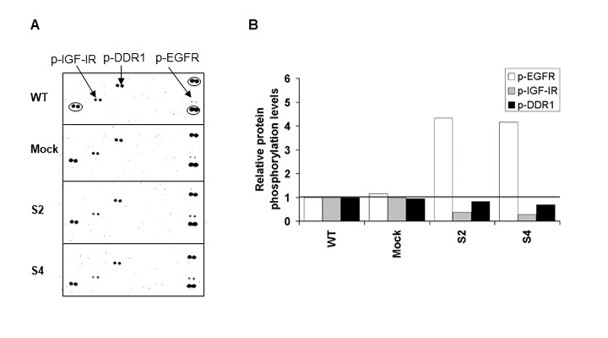


**Supplementary Figure 10. Changes in phosphorylation of EGFR, IGF-1R and DDR1 in *PHLDA1*-silenced human neuroblastoma IMR-32 clones.** The phosphoproteome profiling antibody arrays. Reference proteins on the array are presented in circles (A). Identified tyrosine kinase receptors levels determined with the antibody array in a single experiment using proteins isolated from the IMR-32 cells at 48 h of culture. S2 and S4 – clones of IMR-32 cells transduced with shRNA PHLDA1 lentiviral particles, Mock – clone of IMR-32 cells transduced with control lentiviral particles, WT – IMR-32 cells non-transduced with lentiviral particles. Protein levels for WT set as 1 and marked with the black line (B).


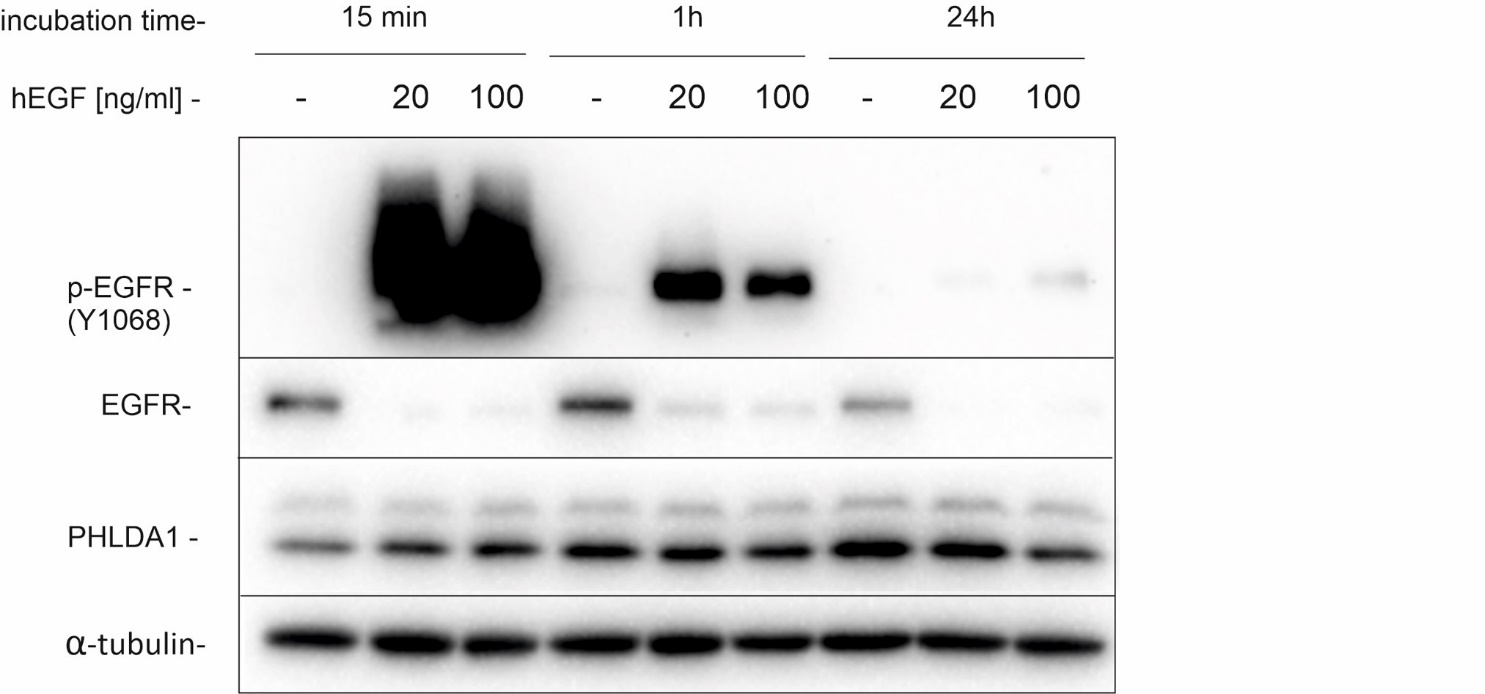


**Supplementary Figure 11. Stimulation with hEGF does not affect PHLDA1 protein level.** IMR-32 cells were stimulated with hEGF at indicated concentrations for 15 min, 1 h or 24 h. Then cells were lysed, and western blot analysis was performed using indicated antibodies. 0.1 % BSA in PBS was used as solvent control. α-tubulin was used as a reference protein. A single experiment was performed.


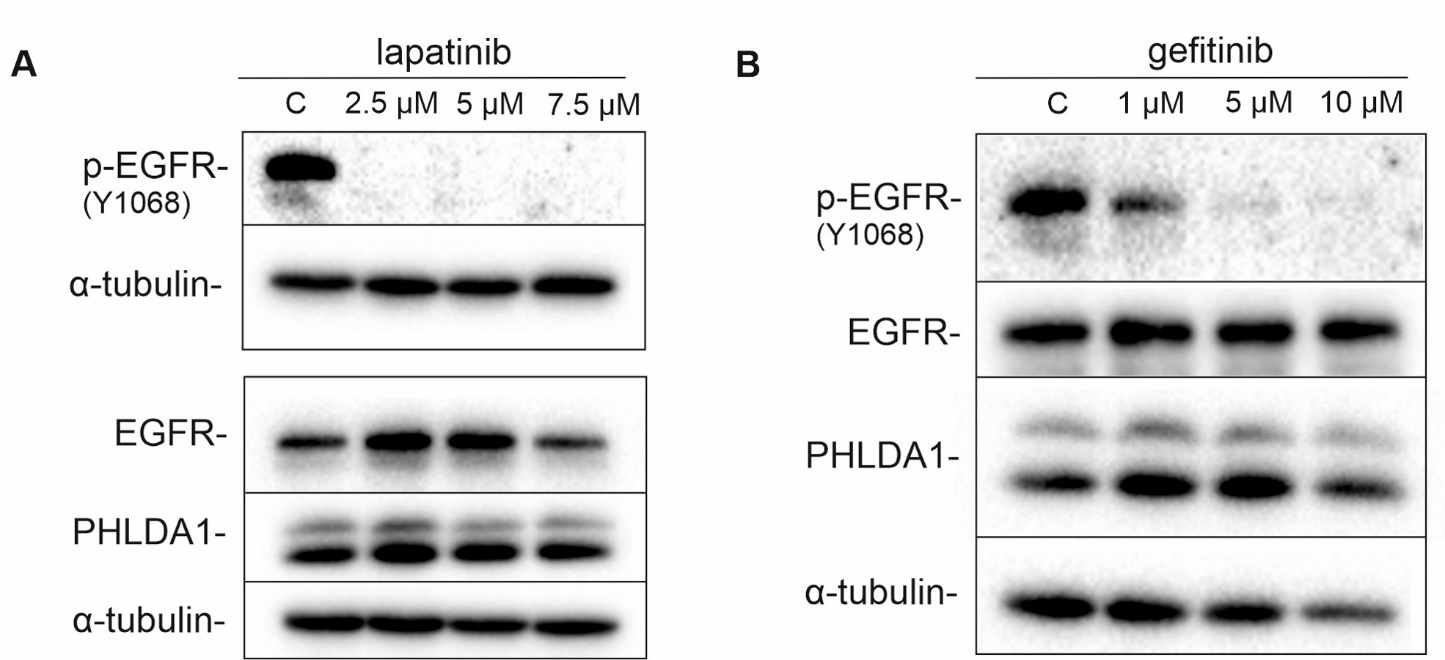


**Supplementary Figure 12. EGFR inhibitors do not affect PHLDA1 protein level.** Unmodified IMR-32 cells were treated with indicated concentrations of lapatinib (A), gefitinib (B) or DMSO as a solvent control and seeded for 72 h. Then the cells were lysed, and the protein levels were determined with western blot using specific antibodies. α-tubulin was used as a reference protein. A single experiment was performed. C – control.


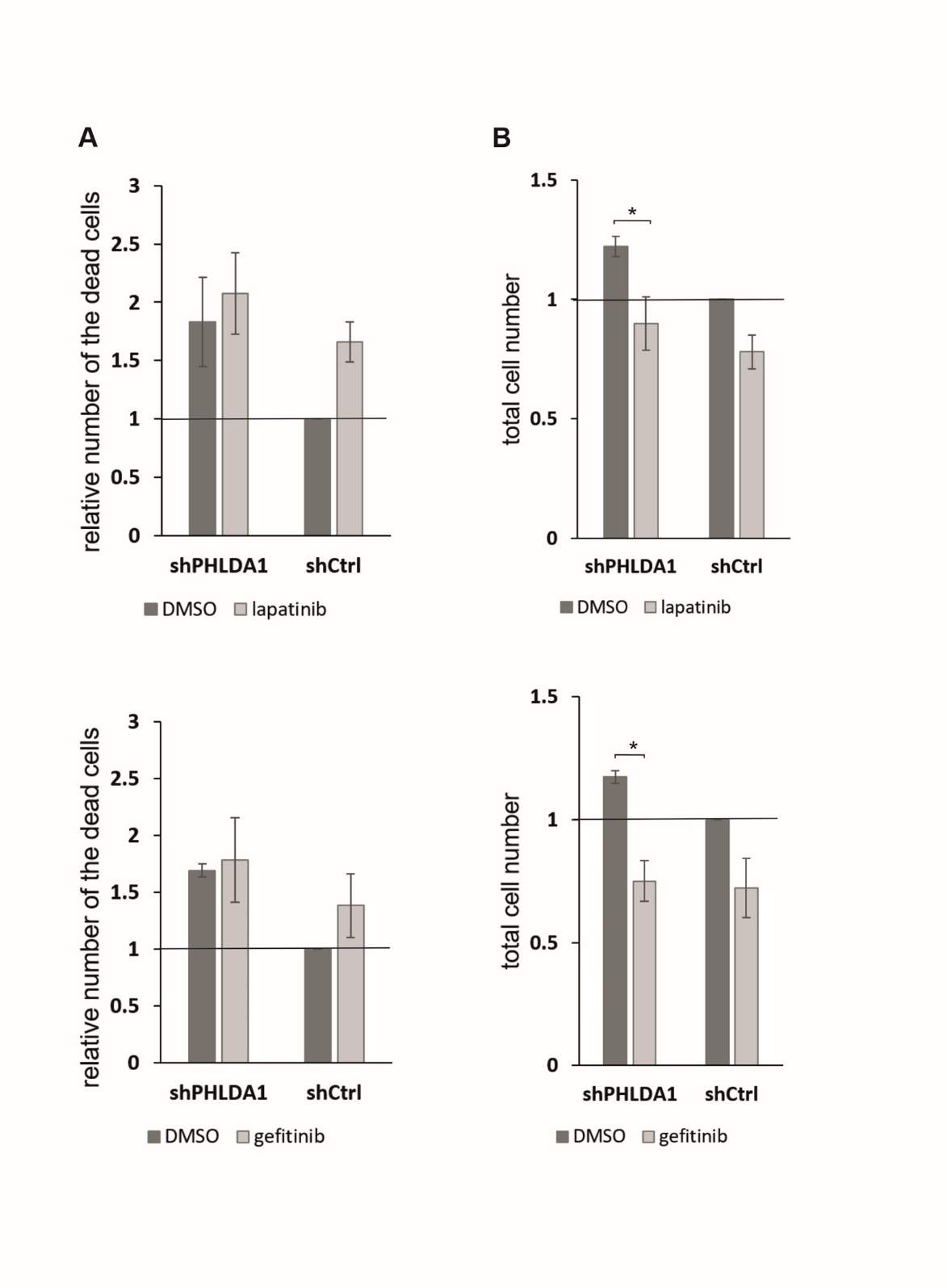


**Supplementary Figure 13. Inhibition of EGFR signaling pathway decreases total cell number in *PHLDA1-* silenced IMR-32 neuroblastoma cells but does not affect the relative number of the dead cells.** *PHLDA1*-silenced (shPHLDA1) and control (shCtrl) cells were treated with 5 μM gefitinib/lapatinib or DMSO and seeded for 72 h. Then cells were stained with trypan blue and counted. Relative number of dead (A) and all (B) cells was compared to DMSO-treated shCtrl cells (set as 1). Data are presented as a mean (+/- SEM) of three independent experiments. Statistical significance was determined by two-way ANOVA with the *post-hoc* Tukey test (*p<0.05).


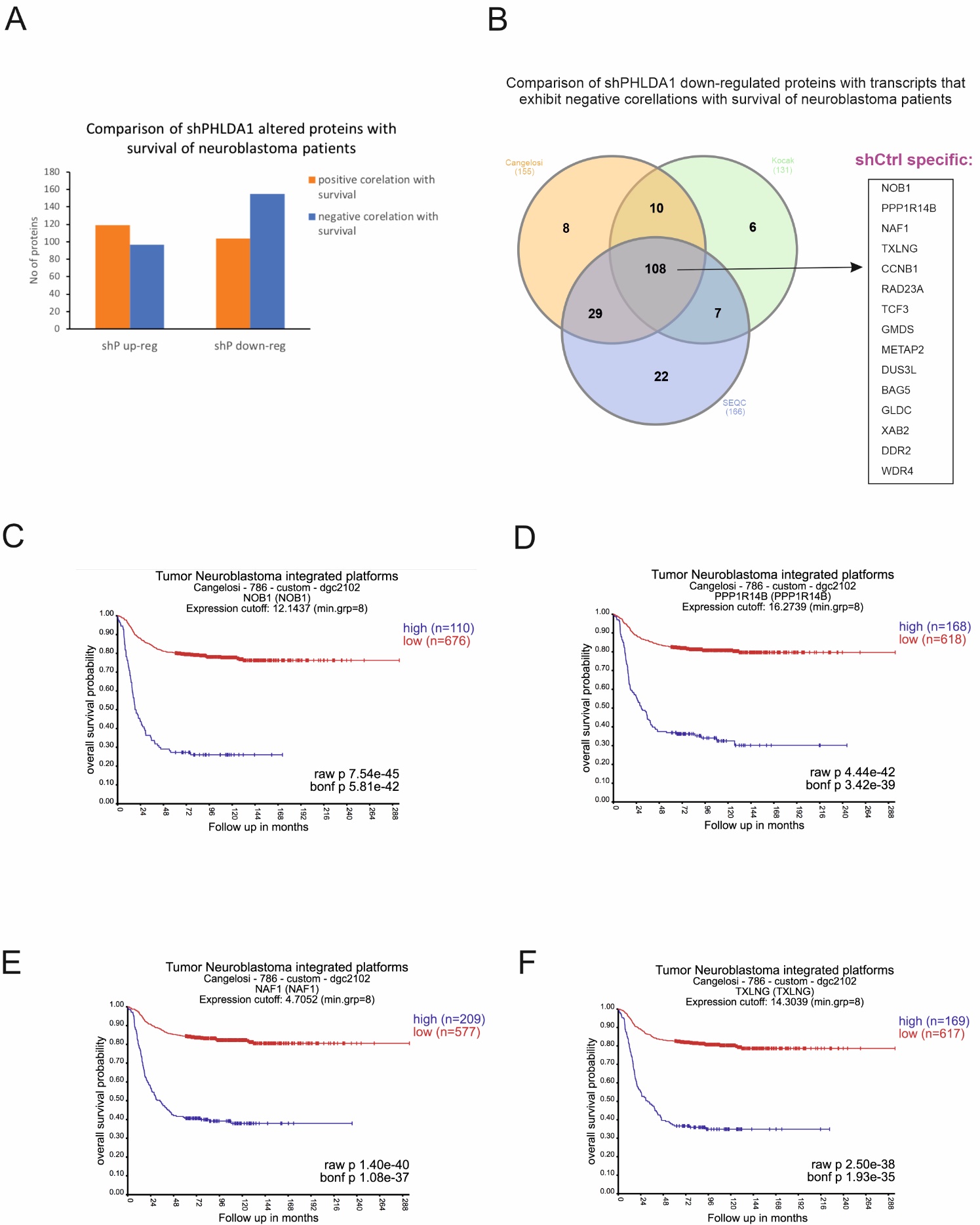


**Supplementary Figure 14.** **Proteins regulated by PHLDA1 might be promising new targets in neuroblastoma.** Bioinformatic analysis was performed by using tools implemented in R2 (http://r2.amc.nl, http://r2platform.com). Expression of significantly up- and down-regulated proteins identified in mass spectrometry analysis after PHLDA1 silencing was compared with overall survival of neuroblastoma patients based on Cangelosi-786 expression dataset available on R2 platform. Number of proteins with significant (p<0.05 with FDR correction) correlation with survival was presented (A). The Venn diagram shows overlapping genes coding for statistically down-regulated proteins, identified in mass spectrometry analysis of PHLDA1-silenced vs control cells, and genes that exhibit significant (p<0.05 with FDR correction) negative correlation of expression with survival of neuroblastoma patients from Cangelosi-786, Kocak-649 (GSE45547) and SEQC (GSE62564) datasets. Overlapping genes were subsequently compared with shCtrl specific proteins and top 15 of identified genes are listed in the right, ordered from the lowest to the highest correlation with survival (B). Kaplan-Meier graphs of correlations between NOB1 (C), PPP1R14B (D), NAF1 (E), TSSC4 (F) mRNA level and overall survival of neuroblastoma patients from gene expression Cangelosi-786 dataset available on R2 platform.


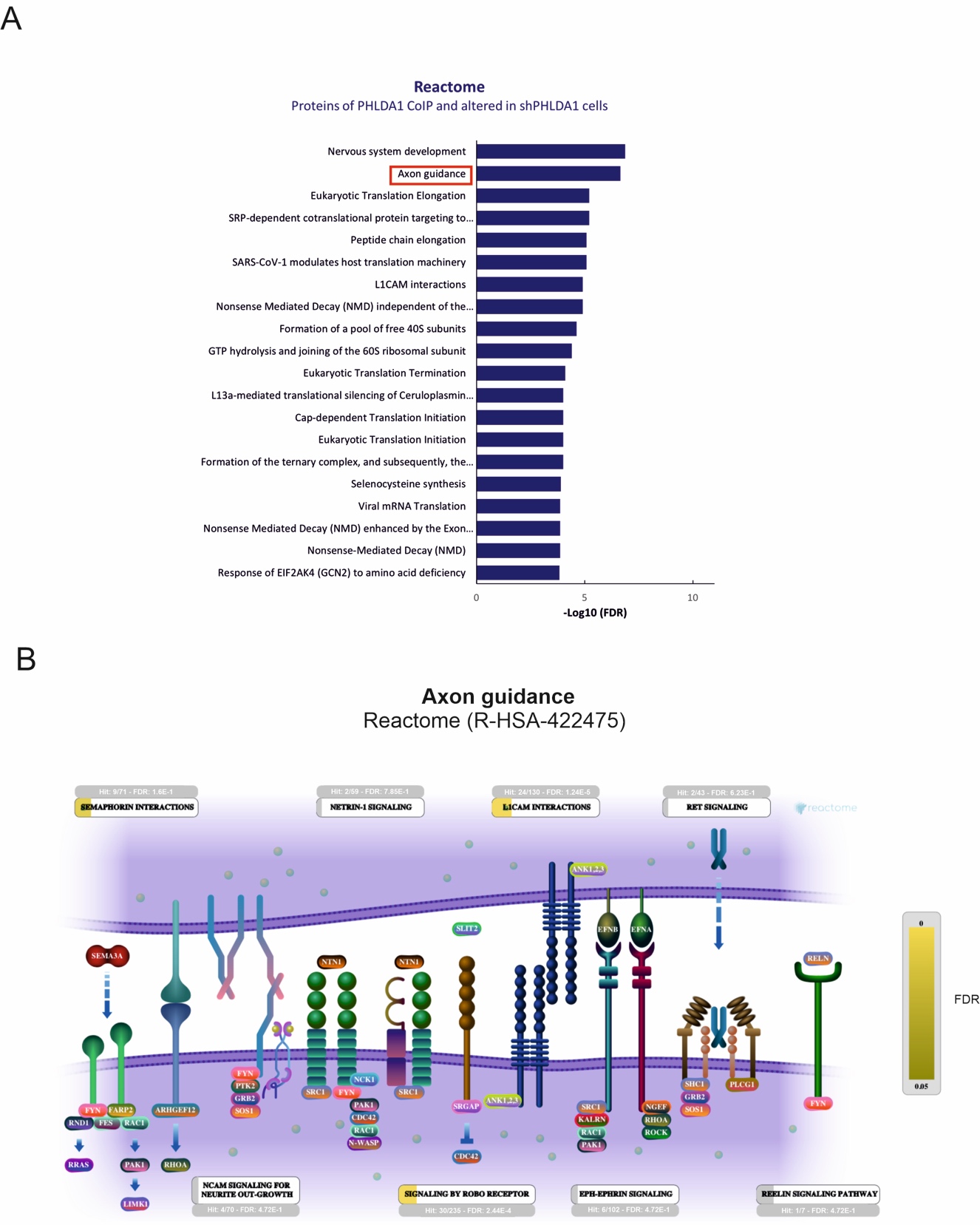


**Supplementary Figure 15. PHLDA1 regulates the axon guidance pathway.** Reactome pathway analysis of combined PHLDA1 binding candidates identified in IMR-32 control cells and proteins which level or post-translational modification status was significantly altered upon *PHLDA1* silencing (A). The modified Reactome axon guidance pathway scheme (R-HSA-422475). Internal signaling pathways enriched in the analysis (within axon guidance) are marked with a number of annotated proteins per the total number of proteins in the pathway and FDR values presented on the grey background above or below of name of the internal pathway (B). FDR - False discovery rate.


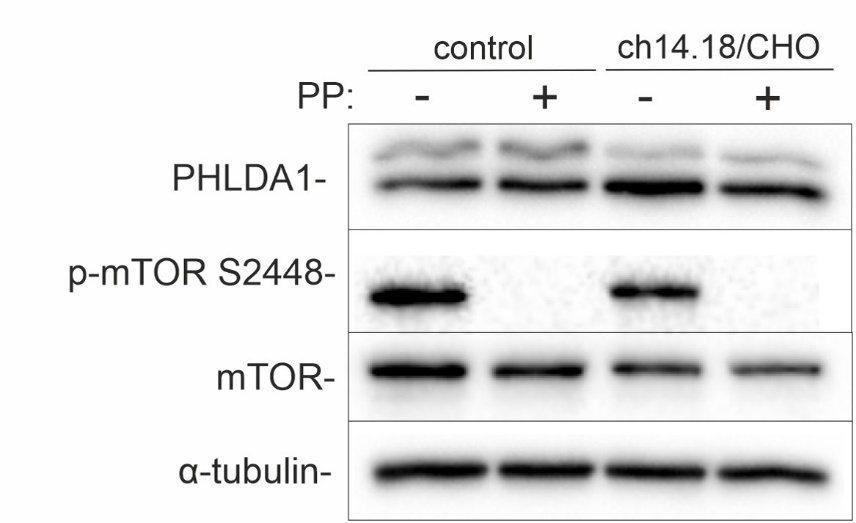


**Supplementary Figure 16. The additional band above PHLDA1 is not a phosphorylated form of PHLDA1.** IMR-32 cells were treated with ch14.18/CHO Abs, at concentration of 40 μg/ml for 48 h. PBS was used as a control. Then the cells were lysed and treated with phosphatase lambda. (+) – cells treated with 600 units/sample of phosphatase lambda and incubated for 1 h in 37 °C, (-) – control cells treated with buffer. Western blot analysis with indicated antibodies was performed. PP – protein phosphatase lambda. Phosphorylated m-TOR was used as a control for PP lambda activity. α-tubulin was used as a reference protein. That experiment was performed once.

## Supplementary Tables

Supplementary Table 1 was already deposited in Frontiers

**Supplementary Table 1. Mass spectrometry data on PHLDA1 binding proteins.** Proteins enriched in one or two replicates in control cells (PBS) or after ch14.18/CHO treatment (AB) are marked with + in columns “Sum enriched”. Proteins enriched in both control and treatment are marked with + in column “Enriched both in PBS and AB”. IgG - CoIP with isotypic control; PH- CoIP with anty-PHLDA1 antibody. Additional sheets “peptide/protein identification” contain detailed MS identification data.

Supplementary Table 2 was already deposited in Frontiers

**Supplementary Table 2. Mass spectrometry data on protein profiles in *PHLDA1*-silenced (shP) and control (shC) cells.** Statistically significant proteins (q value below 0.05) are marked with bold. Significant fold changes exceeding 1.5 are marked with color – red for downregulated, green for upregulated. Proteins specific for one analytical group (0 or 1 LFQ value in one group + at least 3 LFQ values in second) are specified in separate spreadsheets.

Supplementary Table 3 was already deposited in Frontiers

**Supplementary Table 3. Mass spectrometry data on ubiquitination of proteins in *PHLDA1*-silenced (shP) and control (shC) cells.** Significantly changed ubiquitination sites (q value < 0.05 or present only in one group) are presented in bold. Sites marked with color are upregulated in shP.

Supplementary Table 4 was already deposited in Frontiers

**Supplementary Table 4. Mass spectrometry data on phosphoproteome in *PHLDA1*-silenced (shP) and control (shC) cells.** Phosphosites upregulated in shP vs shC cells are presented on yellow background. Changes of proteins that were statistically significant (p<0.05) were bolded.

Supplementary Table 5 was already deposited in Frontiers

**Supplementary Table 5. Detailed protein identification and quantification (LFQ values) data for shP and shC cells.**

Supplementary Table 6 was already deposited in Frontiers

**Supplementary Table 6. Detailed peptide data from mass spectrometry measurements for shP and shC cells.**

Link to Table S7: <https://files.bio.edu.pl/s/SnbcA8n3wCRFRwf>

Direct to file: <https://files.bio.edu.pl/s/SnbcA8n3wCRFRwf/download/Table%20S7.xlsb>

**Supplementary Table 7. Detailed peptide fragmentation data from mass spectrometry measurements of shP and shC cell samples.**

**Supplementary Table 8. Antibodies used in the study.**
